# Wind and solar resource assessment and prediction using Artificial Neural Network and semi-empirical model: case study of the Colombian Caribbean region

**DOI:** 10.1016/j.heliyon.2021.e07959

**Published:** 2021-09-08

**Authors:** Oscar Churio Silvera, Marley Vanegas Chamorro, Guillermo Valencia Ochoa

**Affiliations:** Universidad del Atlántico, Facultad de Ingeniería, Carrera 30 Número 8 - 49, Puerto Colombia, Área Metropolitana de Barranquilla, Colombia

**Keywords:** Renewable energy sources, Solar radiation, Bird & Hulstrom model, Wind speed, Wind power, Back propagation, Artificial Neural Network

## Abstract

This work is focused on the importance of developing and promoting the use of wind and solar energy resources in the Colombian Caribbean coast. This region has a considerable interest for the development of solar technology due to the available climatic characteristics. Therefore, a detailed solarimetric analysis has been carried out in the department of San Andrés, Providencia and Santa Catalina, located in the Colombian Caribbean region, using a semi-empirical radiation model, based on the Bird & Hulstrom model, and the parameterizations of the Mächler & Iqbal model, which allowed obtaining an average total irradiation value of 6.5 kWh/m^2^day. In addition, a statistical analysis of the wind resource was carried out based on meteorological data, which yielded an average multiannual wind speed of 3.4 m/s, and a maximum wind speed of 15.2 m/s during the month of October. The meteorological input data used for this analysis were provided by the Colombian Institute of Hydrology, Meteorology and Environmental Studies (IDEAM), in order to perform initial calculations and obtain a climatic profile of the areas with clear, medium and cloudy atmospheres throughout the year. Regarding the comparative study, the analysis was complemented with a prediction of solar radiation using Artificial Neural Networks (ANN), where irradiance could be predicted with a fairly good agreement, which was validated with a Root Mean Square Error (RMSE) of 0.87 using the temperature, the relative humidity, the pressure and the wind speed as the input data.

## 1 Introduction

The global concern is currently focused on strategies to mitigate climate change and its effects, which are framed by the way countries generate, transport and consume their energy resources, since the energy sector produces a large amount of greenhouse gas emissions [1]. Worldwide awareness is currently focused on strategies to mitigate climate change and its effects, which are framed by the way in which countries generate, transport and consume their energy resources, since the energy sector produces a large amount of greenhouse gas emissions [2], as energy demand is increasing by approximately 5% per year in developing countries, such as Colombia [3].

The ineffectiveness of mega-hydro plants to meet the needs of cities in times of energy shortages, combined with dynamic changes in oil prices [4], as is the case of Floridablanca in Colombia [5], has targeted several studies on the evaluation of wind energy resources as energy supply, under economic viability criteria for decision making in electricity markets [6]. Considering the principles that affect the varying impact on the electricity sector development, through investment models that guarantee the viability of the projects [7], and considering the multiple factors that introduce uncertainty in the calculation to estimate the power generation based on wind and solar resources [8], allows to deal with new energy challenges that allow to control the limitations of traditional methods of valuation of investments in the electricity sector, thus adapting to environmental change, and exploring real methods in which intangibles and operational flexibility are considered as elements that can alter the decision to carry out a renewable energy project [9] offering renewability and cleanliness as two advantages over conventional energy [10].

The Colombian Caribbean region is currently considered one of the largest sources of wind and solar potential at nationwide level, due to the progress that this region has made in recent decades in terms of the implementation of renewable energy sources [11] considering the growing importance that solar and wind energy have been acquiring in the national and global energy matrix [8, 12]. This has awakened the interest of different sectors focused on developing projects that allow the country to take full advantage of its energy resources, making the Colombian Caribbean an attractive sector for local and foreign investors [13].

Based on this need, several studies have been focused on the prediction of solar irradiation in different geographical areas, in order to know the solar potential in each of them [14]. In these works, new methods have been proposed for the estimation of monthly mean global solar radiation, such as the use of a regression model. This model uses as input variables the altitude, the latitude, the longitude, the relative humidity, the temperature and the sunlight duration, along with other variables. This model was compared to the models available in literature, achieving an r^2^ of 0.9425 [15]. Moreover, it was proposed a prediction model, which seeks to identify the relationship between solar radiation and meteorological variables. This work was based on the use of the variable temperature to estimate the amount of energy per unit area at a certain point of the earth. From this model, it was possible to obtain an r^2^ of 0.87, compared to the real measured data [16].

On the other hand, machine learning model implementations have been proposed. One of these proposals focused on the calculation of the solar potential for a residential building. For this, it was sought to calculate the Direct Solar Irradiation (DNI) and the daily Global Radiation (GR), through the implementation of an ANN, in which meteorological, astronomical and radiometric data were used as input variables. From this implementation, it was obtained a r^2^ 0.9918 and 0.994 for the GR and DNI, respectively [17]. Similarly, in Greece, data such as air temperature, radiation, humidity and wind speed have been used to feed an ANN. This network has as objective the prediction of solar radiation, which was validated with the solar radiation data obtained by the empirical method proposed by Hargreaves [18], obtaining as a result an r^2^ that oscillates between 0.6 and 0.82 [19].

One of the main contributions of this research is to quantify and to characterize the solar and wind energy potential in the department of San Andrés, Providencia and Santa Catalina using the Bird & Hulstrom model and parameterizations of the Mächler & Iqbal model. In addition, this research proposes a methodology to quantify the solar potential, by implementing an Artificial Neural Network (ANN), which allows to establish the possibility of implementing clean energy [20] and thus contribute to the national government's approach to use renewable energy sources to meet the country's energy demand. This article presents the information distributed as follows. In the methodological part (section 2) was considered and described all the information related to the Current status, to the Solar potential evaluation models used for the calculation and to the prediction of the analyzed variables. In section 3, the results and analysis of the data are presented, which is the calculation of the solar radiation, data prediction from the neural network and statistical analysis of the distribution. Finally, in section 4 are the main conclusions and remarks.

## 2 Methodology

**Figure 1:**
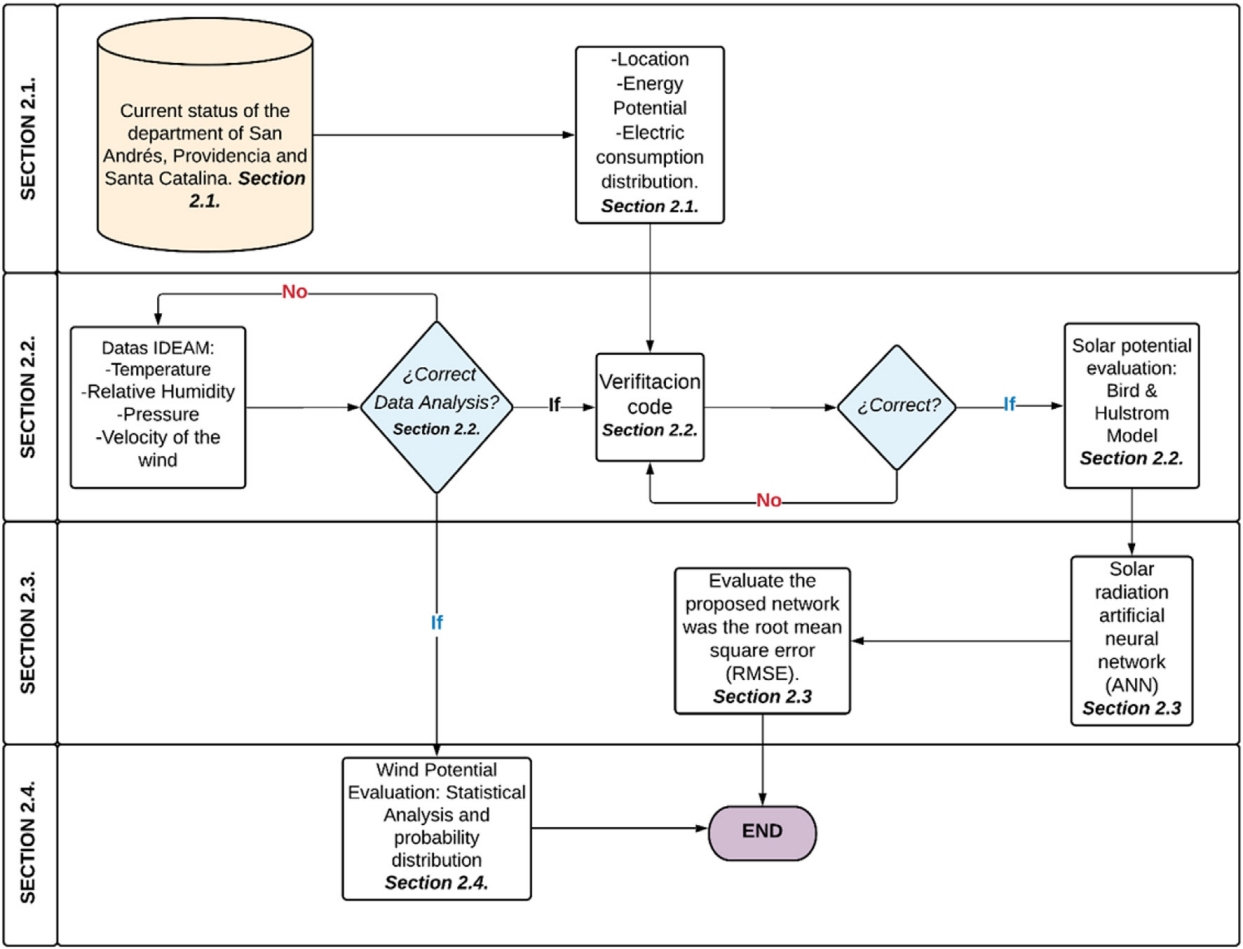
Flowchart of the methodology wind and solar resource assessment and prediction using Artificial Neural Network (ANN).

This research is focused on a solar energy case study located in the Archipelago of San Andres and Providencia, by analyzing the current state of the islands through the Bird & Hulstrom model. Moreover, the prediction of solar radiation is validated by means of an Artificial Neural Network (ANN) in order to validate the behavior of the data and to determine the Mean Square Error (RMSE), using variables such as the temperature, the relative humidity, the pressure and the wind speed as input data. Additionally, the probability distribution equation used to develop the wind potential analysis and the data analysis procedure of the meteorological information is also provided by IDEAM. This methodology is presented in Figure 1.

### 2.1 Current status of the department of San Andrés, Providencia and Santa Catalina

**Figure 2:**
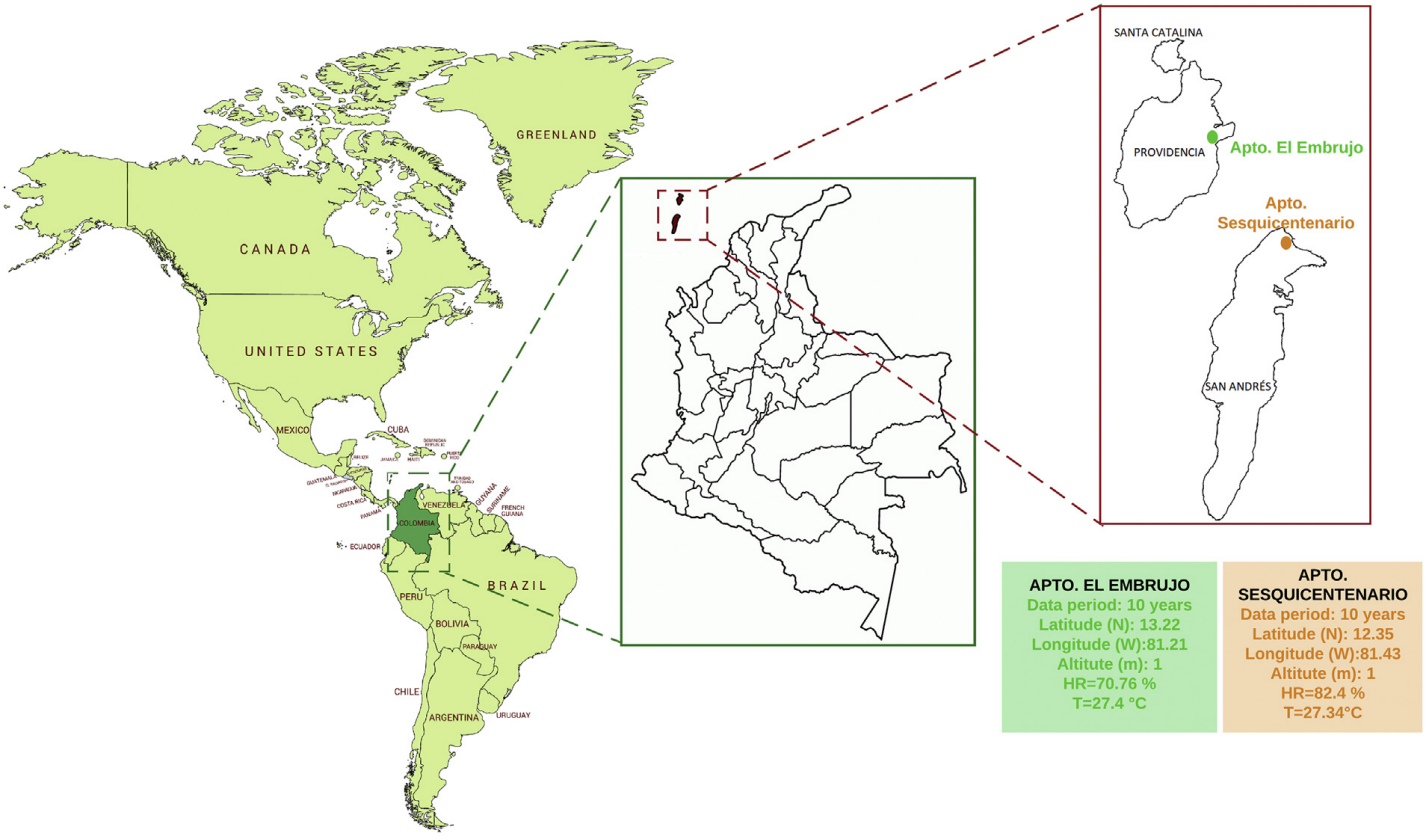
Geographical location of the Archipelago of San Andrés, Providencia and Santa Catalina.

It is very important to know the study area to determine the geographical and meteorological factors involved in the quantification and the characterization of solar radiation. In this particular area there are two meteorological stations (See Figure 2).

The Archipelago of San Andrés, Providencia and Santa Catalina is the Colombian main island territory, both in terms of population and geographic extension. The Archipelago is located in northwestern Colombia at coordinates 12° 35′ 37″ and 14° 42′ north latitude and 81° 40′ 49″ and 81° 43′ 13″ west longitude, entering the central-western sector of the Antilles Sea [21].

Due to its location in the intertropical zone, there are temperatures that register an annual average of 27.3 °C (81.14 **°**F). The influence of the trade winds, which blow from the northeast, determines in part the rainy seasons that begin in the month of May, and reach their peak in the months of October and November extending until December.

Regarding the current state of the Archipelago's electrical system, it should be noted that the provision of electric power is supported by a concession contract in the exclusive service area signed between the Ministry of Mines and Energy of Colombia and the private operator SOPESA [22], which manages all operations, including the generation, distribution and marketing of electricity in the Archipelago. In terms of energy generation, San Andrés has an installed power of 83.6 MW distributed among 18 generation units operated with marine diesel, which is transported from the power plant of Ecopetrol in Cartagena (Colombia). The electrical generation is of the order of 200 GWh/year, with a maximum power demand of 31.4 MW for the San Andrés system.

**Figure 3:**
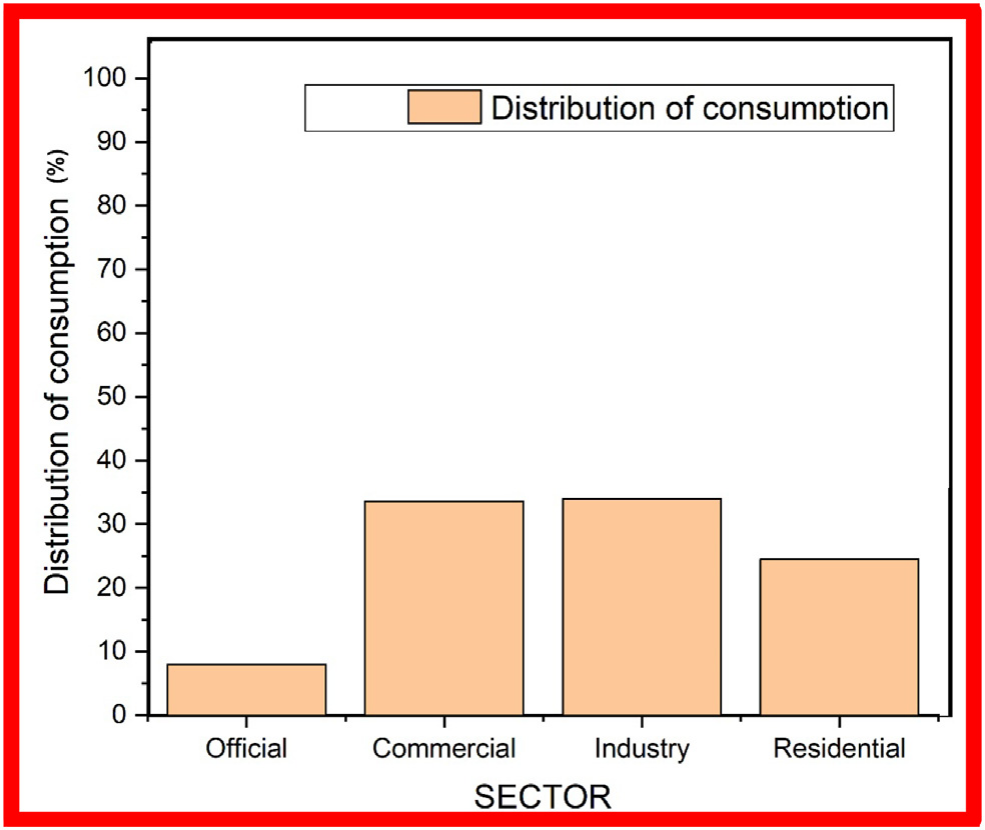
Distribution of electricity consumption by Archipelago Sector Source Unique Information System. (SUI, 2015) for the year 2014 and corresponding to invoiced electricity consumption.

The island of Providencia has an installed capacity of 4.6 MW distributed in four units of between 0.7 MW and 1.4 MW each [23]. The maximum power demand on this island is 1.8 MW and it is estimated that the diesel requirement to operate the Providencia units amounted to 0.8 million gallons in 2013. The electricity consumption invoiced ranges from 155 GWh/year to 165 GWh/year. By comparison, this consumption would represent less than 0.5% of the Colombian electricity consumption if contrasted with the consumption of the National Interconnected System [24]. Consumption is distributed proportionally among the residential, industrial and commercial sectors, each with approximately 30% of total consumption (See Figure 3). Although the hotel sector has less than 0.6% participation by number of users, it consumes a proportion comparable to the other sectors due to the presence of several large hotels in the Archipelago [23].

It is important to be able to guarantee the island's electricity service, quality and energy sustainability in the light of the challenges presented there in the electricity sector.

### 2.2 Solar potential evaluation: Bird & Hulstrom model

This research is focused on a case study on solar energy in the department of San Andres, Providencia, and Santa Catalina, in which the current state of the islands is analyzed, using the Bird & Hulstrom model and parameterizations of the Mächler & Iqbal model with the information provided by IDEAM, organizing and subsequently determining the energy potential under the parameters established by the model to predict the type of solar technology that will be implemented in the studied area.

The Bird & Hulstrom model analyzed the irradiation-direct models of Atwater & Ball and the Hoyt model that constitute a complete proposal of interpretation of the different irradiations on a horizontal surface [25], with the difference that the latter serves to estimate solar global horizontal irradiation and includes a model to determine the direct component [26]. They also interpreted the Lacis & Hansen model that was created to estimate only the global irradiation; however, it served to generate some rigorous spectral codes used as a reference [27] and studied the ASHRAE model. With the ASHRAE model, the global solar irradiation is determined only for the northern hemisphere [28] and finally the Watt model includes the effect of the most common gas mixture on water vapor absorption [29]. On the other hand, there were studies of the irradiation models of Atwater & Ball, Watt, Hoyt, Lacis, and Hansen, as well as the Davies & Hay model that was the first to propose a calculation for irradiation diffusion. However, this last one lacked of a method to treat the aerosol transmittance, requiring a table to estimate the molecular dispersion (Rayleigh dispersion) [30]. As a result of the comparison of the previous models, Bird & Hulstrom formulated an additional model in 1981 [31], [32], which they developed by adopting the formalisms of the analyzed models.

This additional model allows the determination of the total irradiation (*I*_*TH*_) as shown in Eq. (1), from the sum of the irradiation direct (*I*_*DH*_) and the irradiation diffuse (*I*_*dH*_) on the horizontal surface for the whole band [31];

(1) $$I_{TH}=\;I_{DH}+\;I_{dH}$$

To calculate irradiation direct (*I*_*DH*_) in W/m^2^ considering different cloudiness indices on a surface, it is calculated from Eq. (2) [33]:

(2) $$I_{DH}=0.9662\cdot C\cdot \tau_{CTA}\cdot Sin\;\left(A\right)$$

where C, corresponds to the solar constant in relation to the Julian day (W/m^2^), A corresponding to the solar altitude angle in degrees, 0.9662 refers to the correction factor adjusted in the wavelengths of 0.3–3 μm of the solar spectrum, $\tau_{CTA}$ is the coefficient of atmospheric transmissibility, calculated from *τ*_*r*_*, τ*_*o*_*, τ*_*g*_*, τ*_*w*,_ and *τ*_*a*_ that are the transmissibilities by dispersion due to air molecules, ozone, miscible gases, water vapor, and aerosols, respectively. The calculation of irradiation diffuse (*I*_*dH*_) is more complex, as its evaluation requires knowledge of the multiple reflections between the soil and the atmosphere. For its calculation, it is necessary to resort to meteorological observations [33], the irradiation diffuse on a horizontal surface depends on the sky clarity index (*k*_*d*_), the solar constant *C* and the solar altitude angle A. This would be given by Eq. (3):

(3) $$I_{dH}=C\cdot k_{d}\cdot Sin\left(A\right)$$

Irradiation diffuse is the sum of three different contributions: irradiation diffuse due to the permanence of air molecules, diffuse irradiation due to the permanence of dust particles (aerosols), and irradiation diffuse by multiple reflections between the soil and the atmosphere, expressed in Eq. (4) [32]:

(4) $$I_{dH}=I_{dr}+I_{da}+I_{dm}$$

where $I_{dH}$ is the total irradiation diffuse on a horizontal surface, $I_{dr}$ is the irradiation diffuse due to dispersion by air molecules (Rayleigh diffusion), $I_{da}$ is the irradiation diffuse due to the presence of aerosols, and $I_{dm}$ is the irradiation diffuse due to surface reflection. The different types of diffuse irradiation are calculated from Eqs. 5, 6, and 7:

(5) $$I_{dr}=\left(0.79\right)\cdot \left(0.5\right)\cdot \left(C_{r}\right)\cdot \left(\tau_{o}\cdot \tau_{g}\cdot \tau_{w}\cdot \tau_{aa}\right)\cdot \left[\frac{\left(1-\tau_{r}\right)}{\left(1-\;m_{a}+{m_{a}}^{1.02}\right)}\right]\cdot \;Sin\left(A\right)$$

(6) $$I_{da}=\left(0.79\right)\cdot \left(F_{c}\right)\cdot \left(C_{r}\right)\cdot \left(\tau_{o}\cdot \tau_{g}\cdot \tau_{w}\cdot \tau_{aa}\right)\cdot \left[\frac{\left(1-\tau_{r}\right)}{\left(1-\;m_{a}+{m_{a}}^{1.02}\right)}\right]\cdot \;Sin\left(A\right)$$

(7) $$I_{dm}=\left[I_{DH}\;Sin\left(A\right)+I_{dr}+I_{da}\right]\cdot \left[\frac{\left(\rho_{g}\cdot \text{ρ}{\text{’}}_{a}\right)}{\left(1-\;\rho_{g}\cdot \text{ρ}{\text{’}}_{a}\right)}\right]$$

where parameters such as transmissibilities due to aerosol absorption (*τ*_*aa*_), which is a function of air mass (*m*_*a*_), and transmittance due to the aerosols (τ_a_) is used for the calculation of the direct irradiation and (*C*_*r*_) Value of daily solar constant (W/m^2^), and surface reflection coefficients (*ρ*_*g*_). The latter value is usually tabulated by the reflection coefficient for different surfaces is 0.15 for asphalt, 0.3 for grass, and 0.35 for concrete [33]. Similarly, it is necessary to calculate the atmospheric albedo, i.e. the amount of reflections between the sky and the ground ($\text{ρ}{\text{’}}_{a}$), which is a function of *F*_*c,*_ where Fc represents the percentage of the energy that approaches the Earth's surface due to dispersion by aerosols [34].

#### 2.2.1 Data analysis

In order to quantify and characterize the solar potential of the department of San Andrés, Providencia, and Santa Catalina employing the Bird & Hulstrom models, there is a record of historical series of relative humidity and temperature on a daily line at 7, 13 and 19 h for the period the ten years of 2 meteorological stations, as shown in Table 1. These historical records were provided by IDEAM and the minimum reading of the stations was five years.

**Table 1:** Archipelago meteorological stations.

	Weather station 1	Weather station 2
IDEAM Code	17075030	17015010
Name	Apt. El Embrujo	Apt. Sesquicentena
Placement	Providencia	San Andrés
Latitude [N]	13.21	12.35
Length [W]	81.21	81.42
Elevation [msnm]	3	6
Data period	10 years	10 years

**Figure 4:**
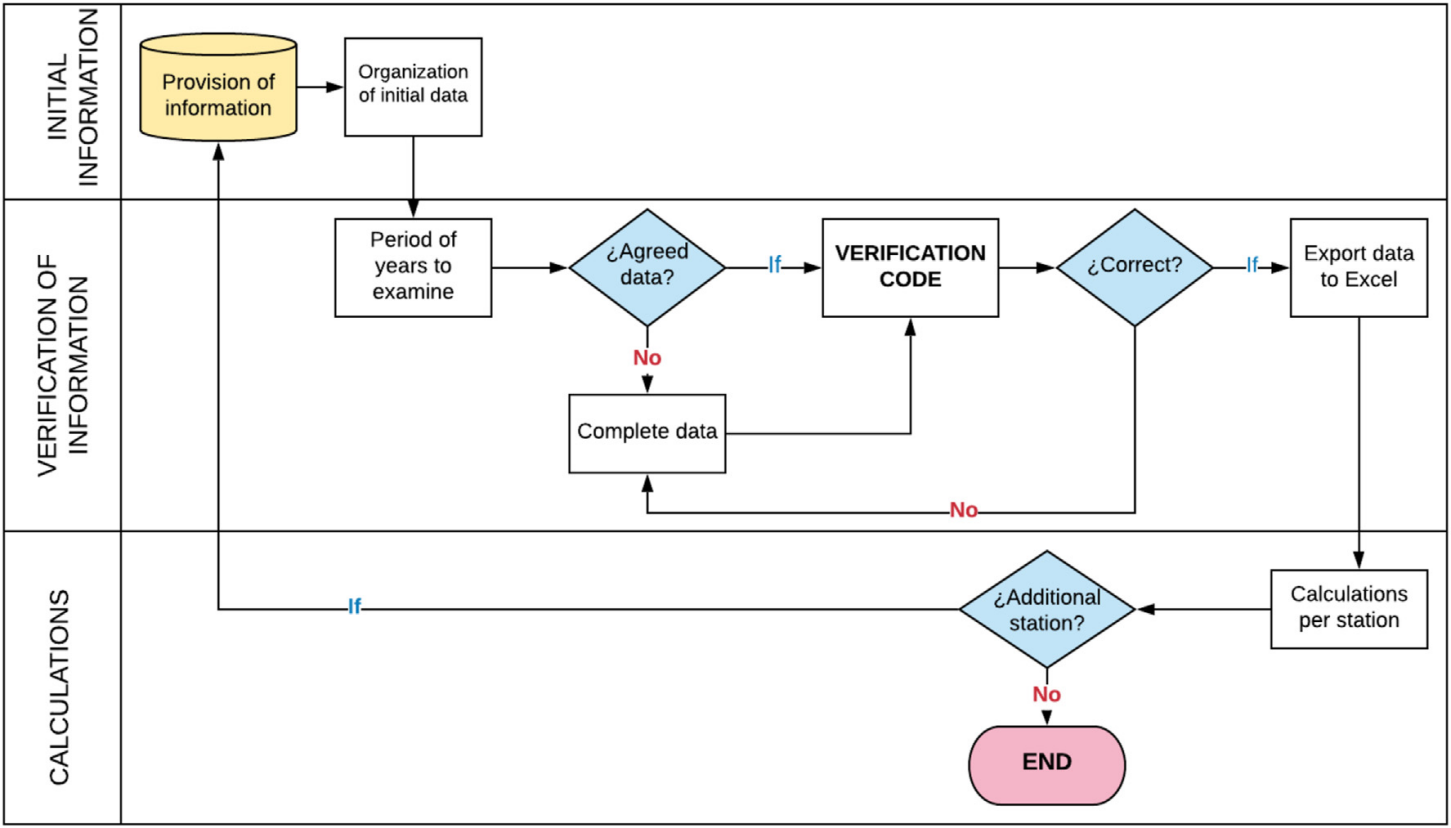
Algorithm for the processing of meteorological data provided.

Through the Matlab® computer tool, a code that allowed a mathematical-statistical treatment was formulated to be carried out in the different time series, lapses, or periods to conduct the information process. This phase of the methodology was necessary due to the fact that the 2 stations in the area, the Apt. Sesquicentenario report missing data in certain years. This as a result of the interruption of readings, failures in measuring instruments, errors associated with changes in measurement conditions, transcription errors, among others (See Figure 4).

### 2.3 Artificial Neural Network

**Figure 5:**
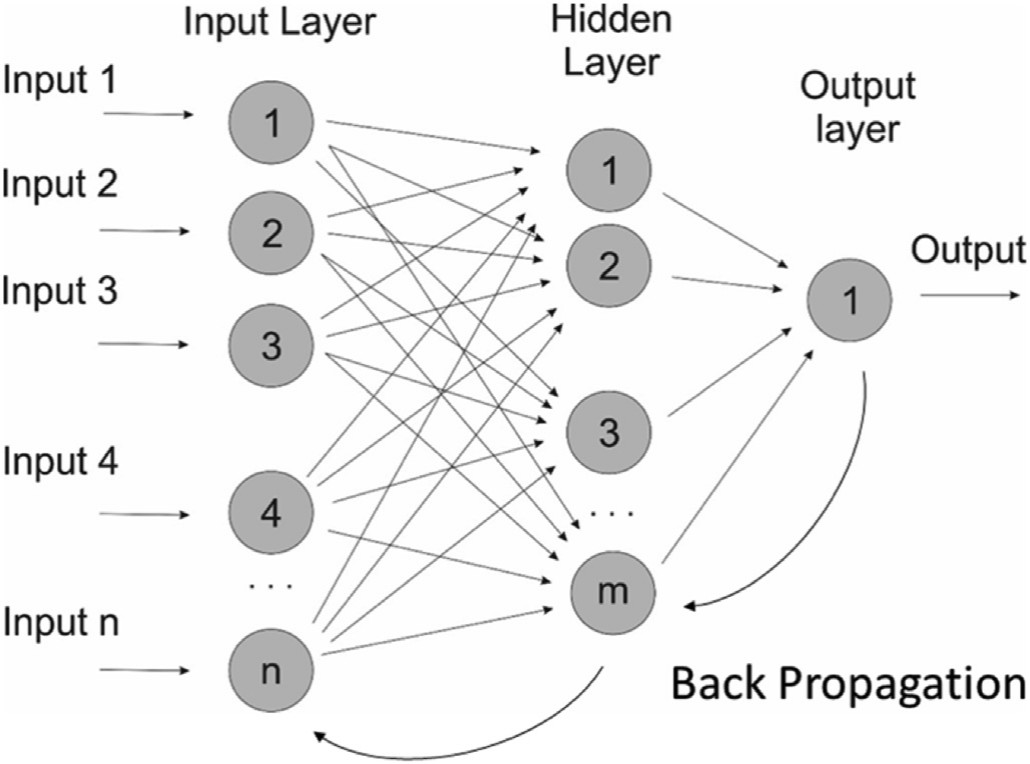
Algorithm for the processing of meteorological data provided b.

The ANN model was used to correlate the inputs and outputs of a phenomena, by mean of a recursive learning from the data. This tool is characterized by learning through training. This knowledge acquired through training is called synaptic weight, and is stored in the strength of the connection between neurons. This modeling method can be applied to phenomena with linear and non-linear relationships [35] (See Figure 5).

To achieve this, each neuron *Y* processes the information, by means of a weighted sum function, taking the values of the previous layer, and the synaptic weight of each connection is taken into account. The behavior of this function *Y* can be represented in a simple way by the Eq. (8).

(8) $$Y_{i}=\;f\left(\overset{N}{\underset{i=1}{\sum }}X_{i}\cdot W_{i}\right)=X_{1}\cdot W_{1}+X_{2}\cdot W_{2}+X_{3}\cdot W_{3}+\ldots +X_{i}\cdot W_{i}$$

where $X_{i}$ is the input value to the neuron, $W_{i}$ is the synaptic weight of each connection and N is the number of connections at the input of a neuron. ANN performance can be evaluated by means of performance indicators, which can be used to calculate the error presented by the model obtained.

In neural network architecture, each node connects to another node in the next neuron, usually making it more convenient to write the retro propagation algorithm in a notation matrix. In this notation, the weight deviation, network input, activation and error signals for all units in a structure are combined as vectors, while the weight adjustment of one structure to the other forms a W-array. The structures are numbered from 0 (input structure) to L (output structure). The retro propagation algorithm is shown from Eqs. (13), (14), (15), (16), (17), and (18) [36]. To start the input structure is based on Eq. (9).

(9) $${\vec{\text{y}}}_{0}=\vec{\text{x}}.$$

Then the activity is propagated forward for i = 1, 2, ..., L, as shown in Eq. (10)

(10) $${\vec{\text{y}}}_{i}={\text{f}}_{i}\left({\text{W}}_{i}\cdot {\vec{\text{y}}}_{\text{i}-1}+{\vec{\text{b}}}_{i}\right)$$

where b_i_ is the weight deviation vector.

The estimation of the error in the output structure is made from that of Eq. (11), while the error retropropagation: for i = L-1, L-2, ..., 1 is made from Eq. (12)

(11) $${\vec{\text{δ}}}_{\text{L}}=\vec{\text{t}}-{\vec{\text{y}}}_{\text{L}}$$

(12) $${\vec{\text{δ}}}_{i}=\left({\text{W}}_{\text{i}+1}^{\text{T}}\cdot {\vec{\text{δ}}}_{\text{i}+1}\right)\cdot {\text{f}}_{i}^{\text{′}}\left({\vec{\text{net}}}_{i}\right),$$

where T is the transposed matrix operator, and $\vec{\text{net}}$ refers to net input which is the weighted sum of outputs of previous neurons. Finally, the updating of the weight and deviation is done with Eqs. (13) and (14)

(13) $$\Delta {\text{W}}_{i}={\vec{\text{δ}}}_{i}{\vec{\text{y}}}_{\text{i}-1}^{\text{T}}$$

(14) $$\Delta {\vec{\text{b}}}_{i}={\vec{\text{δ}}}_{i}$$

In this paper, the error used to evaluate the proposed network was the root mean square error (RMSE), which is calculated using the Eq. (15).

(15) RMSE=(∑i=1N(yi−xi)2)N

where $y_{i}$ is the prediction value, $x_{i}$ is the actual measured value and N is the number of evaluated values. Likewise, the correlation coefficient between the measured values and the predicted values by the proposed network is calculated by the Eq. (16).

(16) R2=[∑i=1N(yi−y¯)⋅(xi−x¯)]{[∑i=1N(yi−y¯)2]⋅[∑i=1N(xi−x¯)2]}12

where $\bar{y}$ is the average of the predicted values, $y_{i}$ is the predicted value, $\bar{x}$ is the average measured value, $x_{i}$ is the measured value and N is the number of evaluated values [37].

### 2.4 Wind potential evaluation: statistical analysis and probability distribution

Statistical tools allow valuable information to be obtained by describing and analyzing the information regarding the speeds of each weather station, as well as by allowing these data to be grouped into classes, in which graphic techniques such as frequency histograms can be developed to show existing patterns in a data set for the analysis of this research. This technique could be used also to validate the measurements by discarding outliers or errors of measurement and the measures in a way that provides a view of the data behavior, allowing to bias them within a classification that will determine the type of analysis to be applied to them.

Data variability is a natural phenomenon in any process which measurement should always be considered as its effects cannot go unnoticed.

One of the variability measures is the range, which is the difference between the largest and smallest value of a sample, regardless of the rest of the observations. However, the variability measure used the most is the standard deviation, which is the measure of the deviation regarding the mean of each of the observations. This measure assumes a positive value when its value is greater than the mean and a negative value if it is less. If the magnitudes of the variations are small, then it can be said that there is little variability because they are close to the mean [38, 39].

They provide information about how data is distributed around central trend measurements, whether it is symmetrical to a vertical axis or whether there is elevation, pointing, or flattening in the center of the distribution, which is evidenced by its graphic representation either through histograms or bar graphs [40].

One of the shape measurements is the bias or asymmetry coefficient that allows the symmetry of the distribution to be measured, determining whether the behavior of a data set on both sides of a central measurement is the same. The most commonly used central tendency is the mean, thus, a frequency distribution is symmetrical with respect to this central value, if for each value of the variable, there is another value with the same equidistant frequency with respect to the mean. In this way, a perfectly symmetrical distribution will have a symmetrical coefficient equal to zero and the mode, the mean, and the median will coincide. When it is greater than zero, the asymmetry will be positive, that is, it is biased to the right, and the mean will be greater than the median, and the median will be greater than the mode. Otherwise, when it is less than zero, it will be biased to the left, the mean will be less than the median and smaller than the mode. A data set is considered symmetrical when the observations fall within the ±5 range [40]. The asymmetry coefficient gis defined by the Eq. (17).

(17) $$g=\frac{\bar{x}-\;M_{o}}{s}$$

where $\bar{x}$ is the mean, $M_{o}$ is the mode, and s is the standard deviation. The wind speed was fitted to four different probability distributions. These distributions were the Gamma, the Gaussian, the Weibull and the Rayleigh, which are presented as follow.

The Gamma probability distribution is commonly used for wind speed prediction. This distribution is characterized by a higher density of values towards the right side of the distribution [41]. The probability density model for this distribution is given by Eq. (18).

(18) $$f\left(x,\alpha ,\beta \right)=\;\left\{ \begin{array}{l} \frac{1}{\beta^{\alpha }\text{Γ}\left(\alpha \right)}x^{\alpha -1}e^{-x/\beta },\;x>0\\ 0 \end{array}\right.$$

where the shape parameter is α, and the scale parameter is β. In the case where α,β>0, the expected mean and variance are: μ=E(X)=αβ, $\sigma^{2}=V\left(X\right)=\alpha \beta^{2}$.

As it was presented in this section related to the frequency distribution, the Rayleigh probability distribution is based on two relevant parameters, α which is the shape parameter, and σ which is the scale parameter. The Rayleigh distribution is adopted when the shape parameter presents a value of 2, and the scale parameter is $\sqrt{2}$ [42], and is calculated as shown as follow in Eq. (19).

(19) $$f\left(x,\sigma^{2}\right)=\;\frac{x}{\sigma^{2}}e^{-x^{2}/2\sigma^{2}}$$

where x takes values less than 0. If the shape parameter takes a value of 3, the distribution to be applied will be the Gaussian one.

This distribution is the most commonly found in the phenomena that are analyzed daily, making it one of the best-known distributions in statistics. For this reason, this probability distribution function is frequently used in physical measurements, using Eq. (20).

(20) $$f\left(x,\mu ,\sigma \right)=\frac{1}{\sqrt{2\pi }\sigma }\exp\left[-\frac{1}{2}{\left(\frac{x-\mu }{\sigma }\right)}^{2}\right]$$

Taking σ as the standard deviation, and −∞<x<∞,−∞<μ<∞, and σ>0.

Weibull probability distribution is the most commonly applied probability density function for wind speed analysis and prediction. Like the Rayleigh distribution, it is influenced by a shape parameter (k or α), and a scale parameter (c, θ or β) [43]. For the case of a random variable x, the probability distribution function is given by Eq. (21).

(21) $$f\left(x;\alpha ,\theta \right)=\left\{ \begin{array}{l} \frac{\alpha }{\theta^{\alpha }}x^{\alpha -1}exp\left[-{\left(x/\theta \right)}^{\alpha }\right]\\ \;0 \end{array}\right.$$

wherex,α,θ>0.

## 3 Results and discussions

Through the studied models, it was possible to quantify and to characterize the solar radiation of the department of San Andrés, Providencia, and Santa Catalina for each one of the atmospheres. Similarly, it was achieved through statistical analysis to determine the wind potential of the study area.

### 3.1 Total radiation

According to what was calculated through Eq. (1) of the model, the total radiation values of the two meteorological stations of the archipelago are remarkably similar (see Table 2, where *β* is the turbidity coefficient of the sky and takes the values of *0.0, 0.1, 0.2, 0.3* and *0.4*). This is mainly due to the semi-humid climate and relief that characterizes the area. It should be noted that even for a turbid atmosphere (*β = 0.3*) and very turbid (*β = 0.4*) the results of total radiation are very appreciable. In the same way, it can be observed that the cleaner the atmosphere the higher the radiation values, this is because there are no particles that attenuate the rays from the sun.

**Table 2:** Average annual total radiation of the archipelago of San Andrés, Providencia, and Santa Catalina.

Stations	Apt. Sesquicentenario [W/m^2^]	Apt. Embrujo [W/m^2^]	Average [W/m^2^]
I_TH_ (β = 0.0)	984.3	985.7	985.0
I_TH_ (β = 0.1)	948.3	949.4	948.9
I_TH_ (β = 0.2)	919.0	919.9	919.4
I_TH_ (β = 0.3)	895.1	985.7	940.4
I_TH_ (β = 0.4)	875.6	876.4	876.0

**Figure 6:**
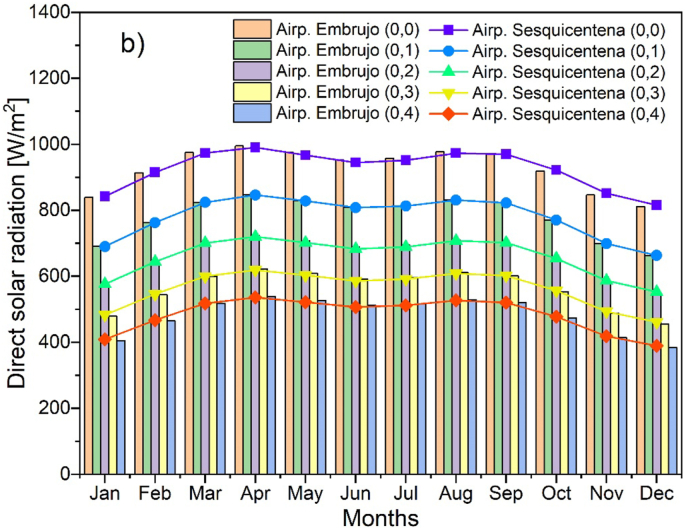
Solar Radiation a) Diffuse, b) Direct of the department of San Andrés, Providencia, and Santa Catalina.

To determine the total solar radiation, it was necessary to quantify the amount of direct and diffuse solar radiation that reaches the area. Figure 6 shows the behavior of direct and diffuse solar radiation from the archipelago's stations and highlights that the months with the greatest radiation in the year are April, May, August and September; and the months with the least solar radiation are January, February, June, November, and December in all types of atmosphere. In other words, solar radiation in the year has a bimodal behavior. Similarly, it is detailed that as the atmosphere becomes turbid the contribution of radiation diffuse increases.

### 3.2 Total monthly irradiation per day

In order to calculate the total irradiation per day, it was necessary to know the behavior of the solar hours of the study area. Sunshine or solar hours is a variable that represents the total time during which direct sunlight falls on a location, between sunrise and sunset, depending directly on the climate of the location [44]. In the behavior of the average monthly sunshine, values of more than 9 solar hours are reached in months such as March and April. In other words, in the first semester of the year, the area has greater solar hours and in the months of the second semester, such as October and November, the values of 6 h are the lowest of the year. This is due to the rainfall generated at that time of year.

**Figure 7:**
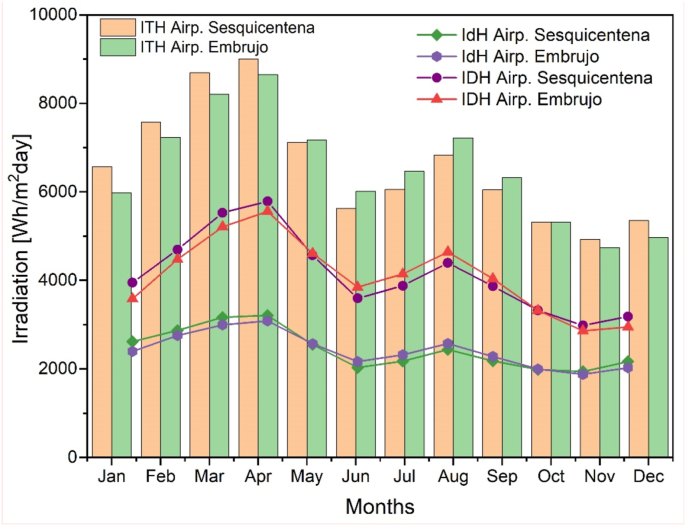
Average total monthly irradiation per day [Wh/m^2^day] at weather stations in the departments of San Andres, Providencia and Santa Catalina.

**Figure 8:**
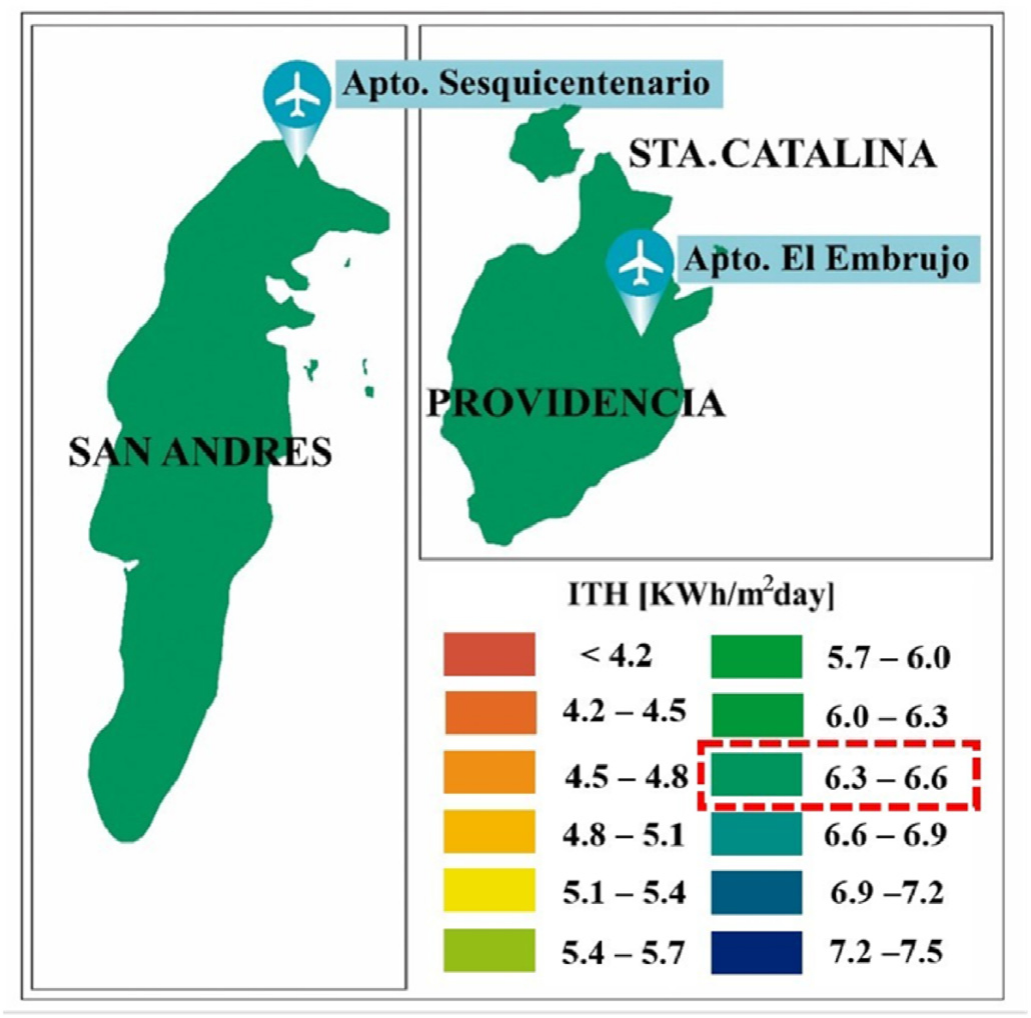
Average total annual solar irradiation map ITH [kWh/m^2^day] of the departments of San Andrés, Providencia, and Santa Catalina.

The months with the highest hours of sunshine are the months with the highest values of total monthly radiation per day, as shown in Figure 7. When considering a cloudy atmosphere (*β = 0.3*), the solar irradiation values show a bimodal behavior that reaches higher averages of 7,0 [kWh/m^2^day] in the months of March and April, while the minimum values are presented in the months of June and between October and December. On average, the irradiation that reaches the departments of San Andrés, Providencia, and Santa Catalina is 6.5 [kWh/m^2^day] (See Figure 7 and Figure 8), an optimal result for the development of new solar technologies in the area of study.

### 3.3 Predicting solar irradiation through the Artificial Neural Network (ANN)

Temperature, relative humidity, wind speed, wind direction and pressure data were used as input for the proposed ANN. These data were taken in 10000 measurements, which were performed over a period from 1980 to 2014. Of these data, 8000 were taken for network training and 2000 for network validation. To these data, a training was made, having as reference the calculated value. In this training the parameters were varied, in search of the most appropriate combination for the proposed case. In Table 3 the results of this training are presented for values of β from 0.0 to 0.4, together with the variation of the number of layers, number of neurons per layer and the learning rate.

**Table 3:** Results of the RMSE for the different trainings.

β Values	Number of layers	Number of neurons per layer	Learning rate	RMSE
0.0	5	20	0.0001	0.010
0.0	15	10	0.001	0.008
0.1	5	20	0.001	0.010
0.1	15	10	0.001	0.010
0.2	5	20	0.0001	0.010
0.2	15	10	0.0001	0.009
0.3	5	20	0.001	0.010
0.3	15	10	0.0001	0.009
0.4	5	20	0.001	0.009
0.4	15	10	0.0001	0.008

**Figure 9:**
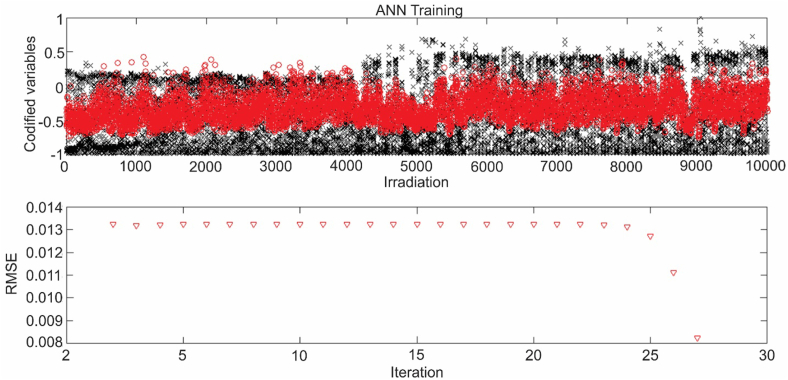
ANN training for β = 0.0.

This table shows that the β values with the best results are 0.0 and 0.4. In which, 15 hidden layers were used, 10 neurons per layer and the activation function was the Rectified Linear Unit (RELU). In the Figure 9 you can see the result of the training for the irradiation values a β = 0.0.

**Figure 10:**
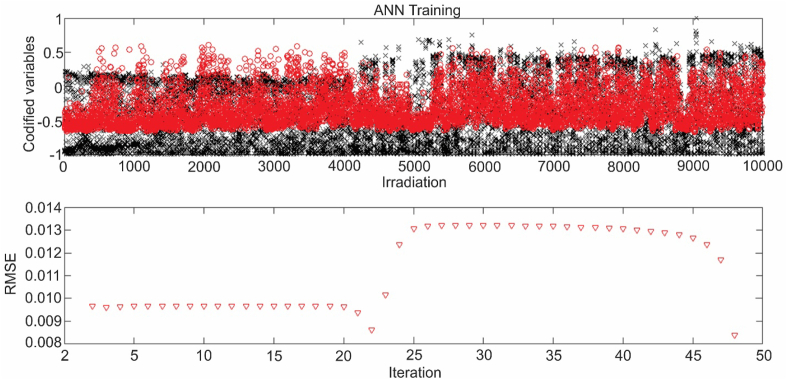
ANN training for β = 0.4.

In this training, a rapid decrease in the RMSE is observed as of iteration 25, reaching the maximum permissible error of 0.008, which was set as the convergence criterion for ANN training. Similarly, Figure 10 shows the training behavior of the network carried out for irradiation calculated with a β of 0.4.

**Figure 11:**
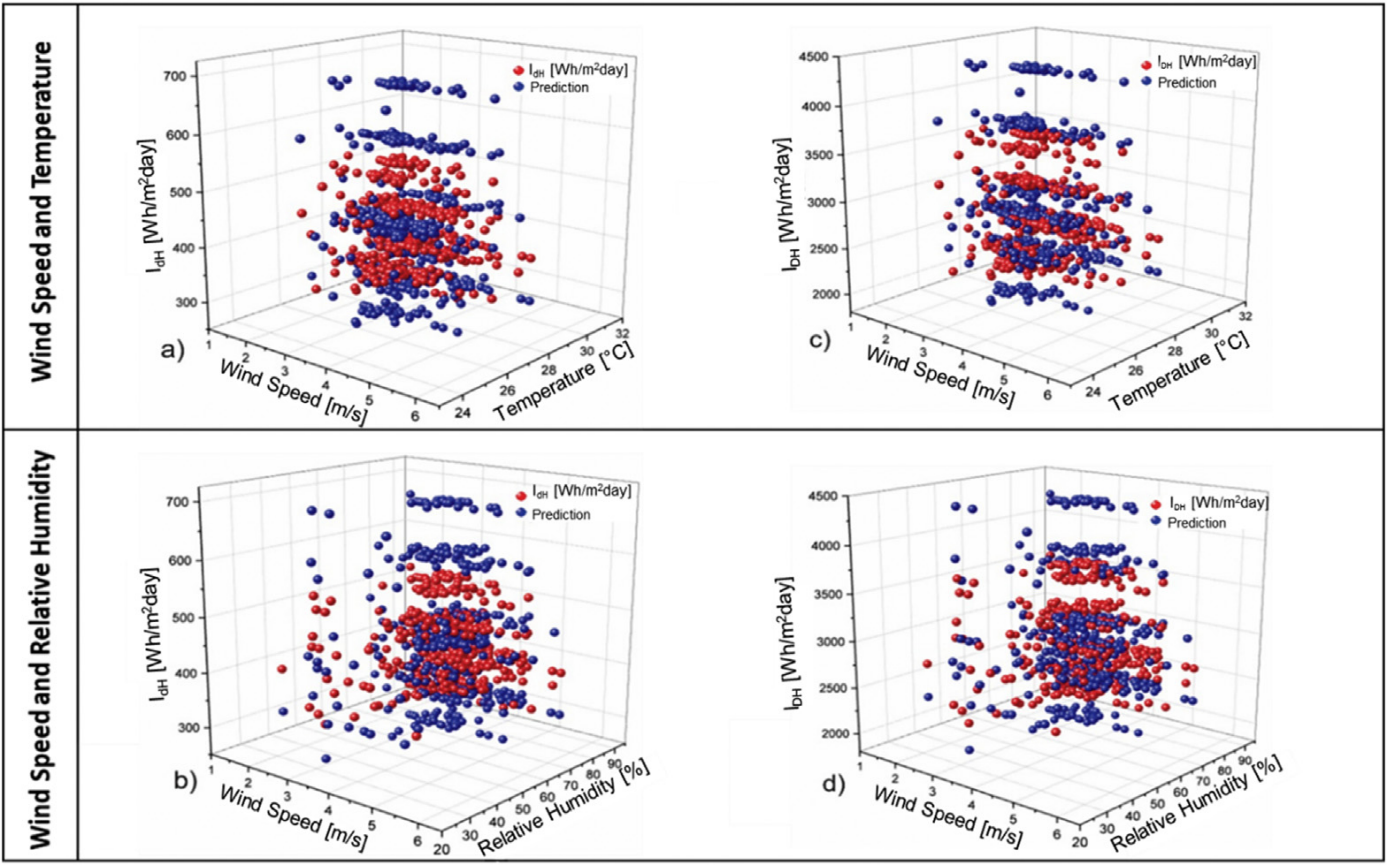
ANN validation for a-c) wind speed and temperature, b-d) wind speed and relative humidity.

Unlike the behavior of the previous training, the RMSE did not reach the convergence criterion in the first fall of the value. This is due to the nature of the method of initiation of the weights, which takes random weights, which can generate a greater or lesser number of iterations. After obtaining the ANN for the two cases studied, a validation of the results of the prediction obtained by means of this was carried out. This validation is presented in Figure 11, where the results of irradiation are observed, having as input data the wind speed, temperature and relative humidity. In Figure 11a, the irradiance for wind speed and temperature is presented using 0.0 as the value of β. For this combination, it is observed that the predicted data cloud has a good adjustment against the real ones when the irradiance values 350 and 500 Wh/m^2^day.

On the other hand, Figure 11b shows the behavior of the irradiance data for wind speed and relative humidity, where a related adjustment is observed in the same range as in Figure 11a. Similarly, Figure 11c shows the behavior of ANN prediction against the calculated data. It shows a negative behavior of ANN prediction in extreme values. This behavior is repeated in Figure 11d, which shows prediction values greater than 3750 Wh/m^2^ day, which is much higher than the calculated values.

**Figure 12:**
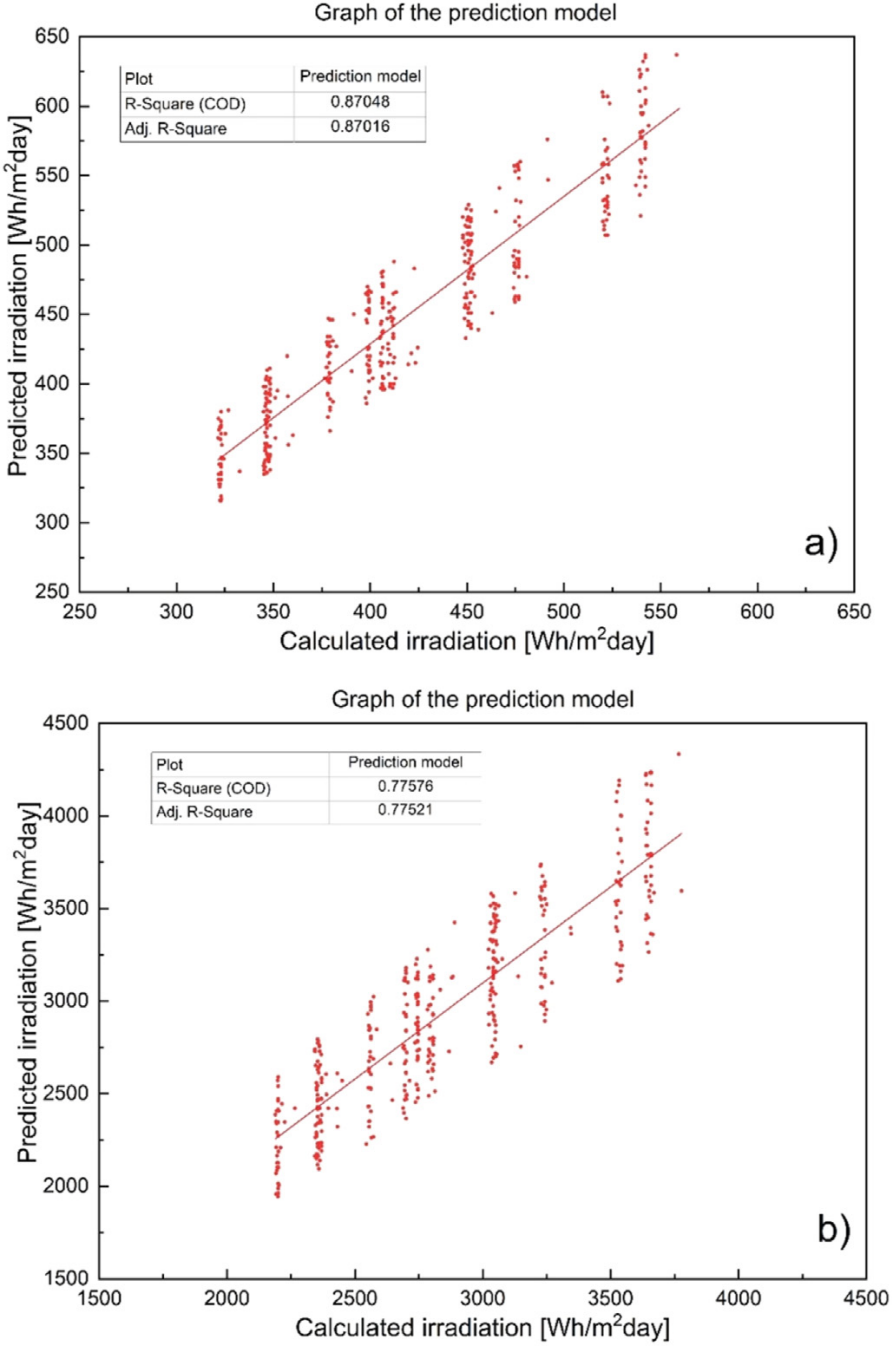
Correlation between calculated and predicted values for a) β = 0.0, and b) β = 0.4.

This validation can be better evaluated by means of the r^2^ presented by the predictions. Figure 12a and Figure 12b presents the results of these for values of 0.0 and 0.4 respectively. It can be seen that the best adjustment of ANN was presented in Figure 12a, in which a β of 0.0 was used, the correlation achieved in this case study was 0.87. This value is higher than that observed in Figure 12b, which represents the correlation between the results of the calculation with a β of 0.4 and the prediction made by ANN.

### 3.4 Wind energy resource in the department of San Andrés, Providencia, and Santa Catalina

Currently, wind energy resources play an important role in the development of renewable energy technologies. By mentioning this resource, direct reference is made to energy obtained through wind kinematics. Wind energy offers enormous potential for the supply of significant amounts of clean electrical energy.

Therefore, this section develops a statistical analysis of the wind speed data related to the stations Apt. El Embrujo and Apt. Sesquicentenario, divided into two parts. The adjusted distributions for monthly data were calculated from a period of ten years, this being the way to identify month by month the greatest wind potential.

The "El Embrujo" station is used as a model station for the analysis of wind speed and direction data, applying in detail the statistical tools described above. Through the statistical study of the data measured in this station, a sample of the result of the wind potential in the Archipelago is obtained. However, the document includes in Appendix A the results obtained in a summarized way for the station Apt. Sesquicentenario.

Table 4, presents the frequencies of the data measured multiannual for the twelve months of the year, calculating the measures of average, standard deviation, bias, and kurtosis; at the same time that the maximum and minimum data for a given month are shown.

**Table 4:** Statistical analysis of the data from the weather station Apt. EL EMBRUJO.

Month	Counting	Average (m/s)	Standard deviation	Coefficient of variation	Min (m/s)	Max (m/s)	Range (m/s)
January	8184	3.7826	1.1925	31.53%	0.1	9.6	9.5
February	7464	3.4309	1.1266	32.84%	0.1	7.8	7.7
March	8184	3.3754	1.1838	35.07%	0.1	7.2	7.1
April	7920	3.1351	1.1466	36.57%	0.1	10.3	10.2
May	8184	3.0934	1.3233	42.78%	0.1	9.3	9.2
June	7920	3.3945	1.4436	42.53%	0.1	9.8	9.7
July	8184	3.7131	1.2403	33.40%	0.1	7.8	7.7
August	8184	2.9892	1.1083	37.08%	0.1	8.5	8.4
September	7920	2.3661	1.1277	47.66%	0.1	7.2	7.1
October	8184	2.4205	1.1953	49.38%	0.1	15.3	15.2
November	7920	3.0595	1.0954	35.80%	0.1	9.3	9.2
December	8184	3.4677	1.2270	35.39%	0.1	7.5	7.4

Table 4 shows the data obtained from wind speed in the different months of the year during the period the 10 years. In this table, it is observed that in months like April with 7920 data, the wind speed goes from a minimum of 0.1 m/s to a maximum of 10.3 m/s. In addition, a standard deviation of 1.146 m/s was estimated. Another month where there is a considerable value of average wind speed is October, where this value was obtained by performing an analysis of 8184 data between 0.1 m/s and a maximum value of 15.3 m/s, this being the month where the average wind speed reaches its maximum value with a data dispersion of 1.195 m/s.

### 3.5 Multi-annual frequency distributions

In different geographical locations, the wind speed presented relevant variations, representing highly significant randomness for the study conducted on the Caribbean coast. The frequency probability distribution of the wind speed allowed to characterize the wind speed at a given location in two ways: first, the frequency probability distribution allows a broad observation of the frequency of the given wind speed; and second, the range of optimum speeds at a respective location.

**Figure 13:**
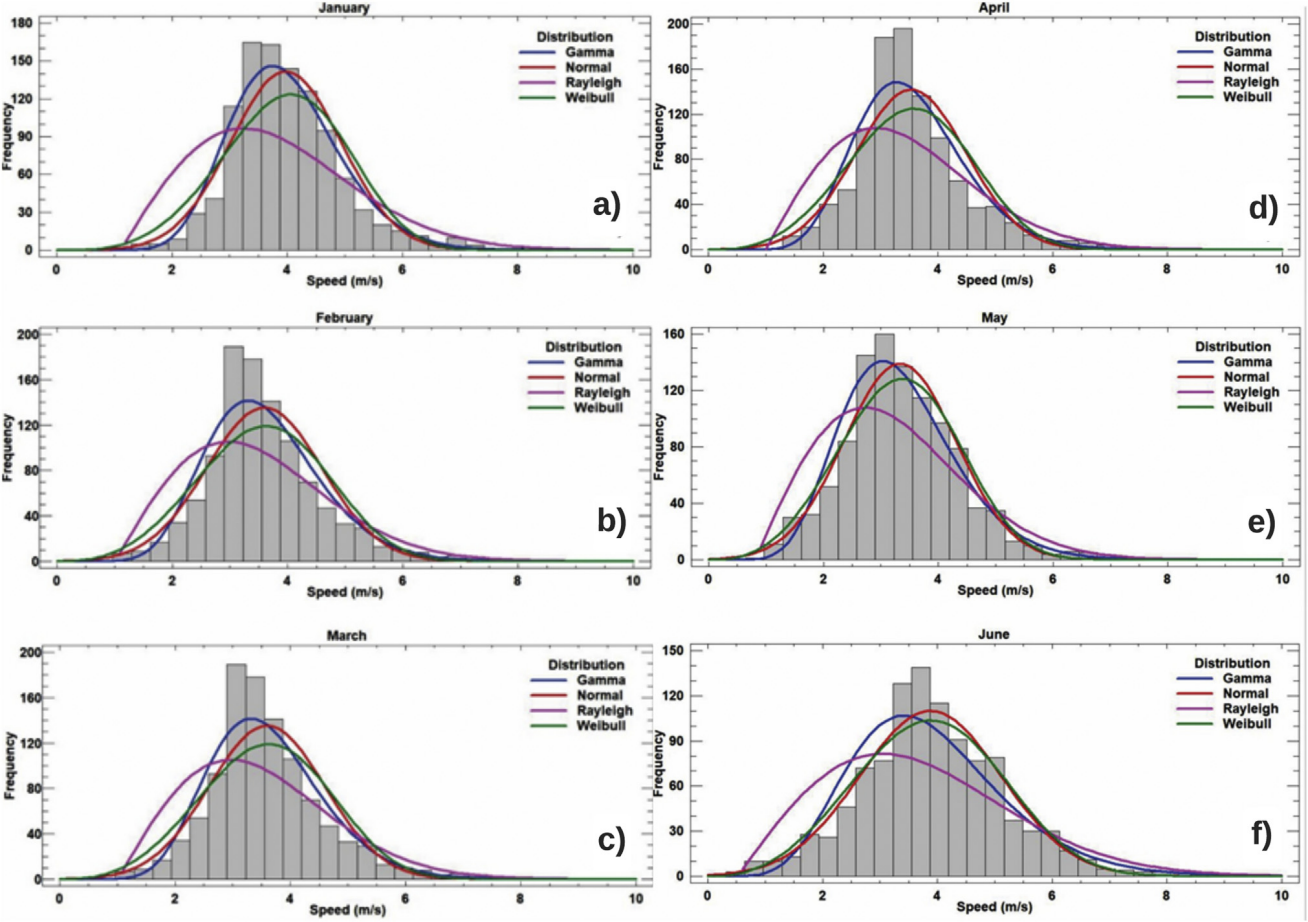
Frequency distribution for wind speed in the months a) January, b) February, c) March, d) April, e) May and f) June.

**Figure 14:**
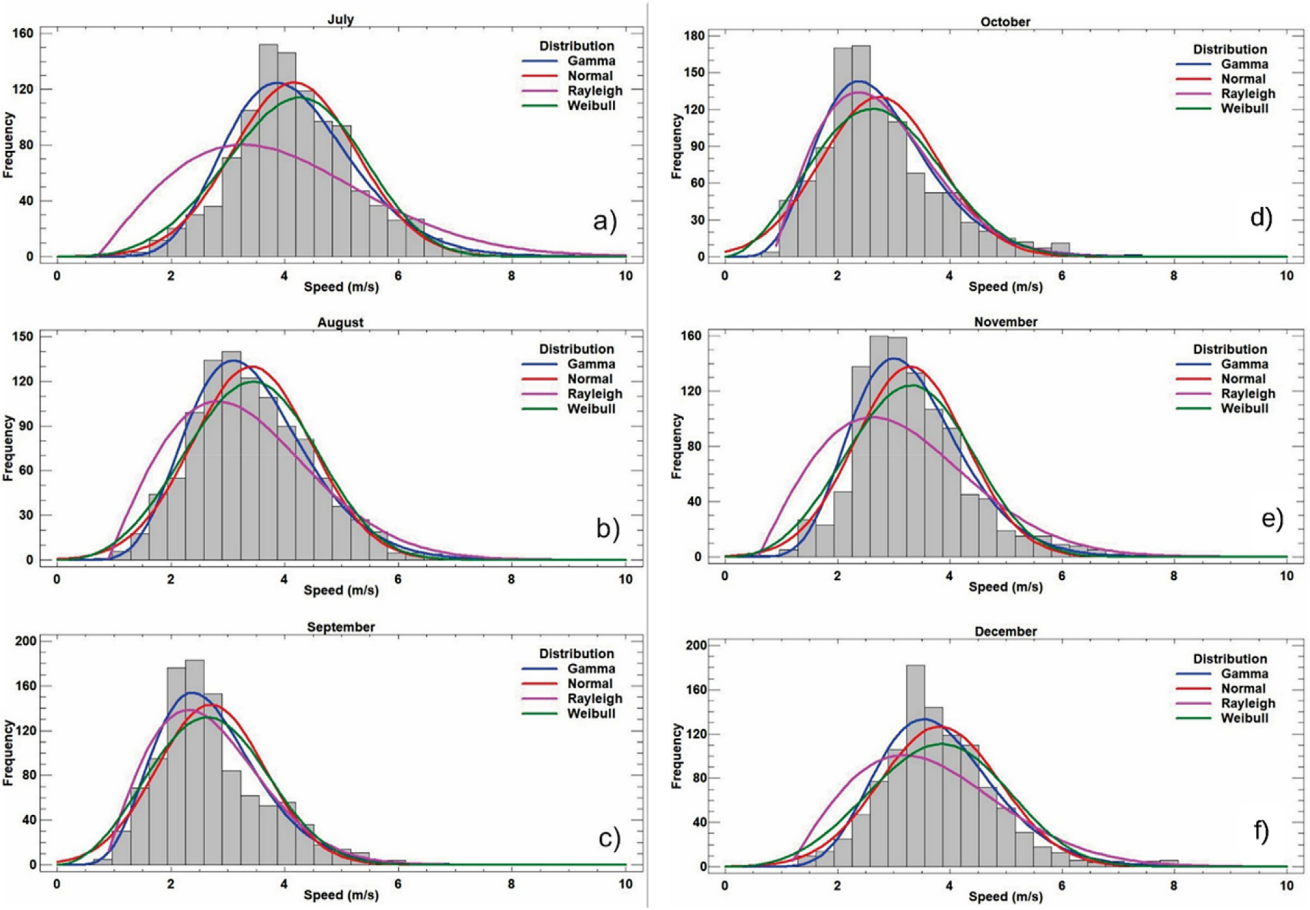
Frequency distribution for wind speed in the months a) July, b) August, c) September, d) October, e) November and e) December.

The above frequency analysis is obtained by ordering the wind speed observations in bins of 1 m per second [m/s] and calculating the percentage of each bin, which allows wind speed to be expressed either by an average value or an instantaneous value. Here is presented, using the normal distributions gamma, Rayleigh and Weibull, the trend of the data of speed and direction of the wind, comparing these statistical distributions it is possible to obtain a validation of the trend of the data by months by season. Each type of distribution is considered to have characteristic values that define the range of values covered by the distribution graph as shown on Figure 13 and Figure 14 for each month, the characteristic values according to each distribution are presented multiannual in Table 5.

**Table 5:** Multi-yearly adjusted distributions for the weather station Apt. El Embrujo.

Month	Gamma		Standard		Rayleigh		Weibull	
January	Form	8.315	Medium	3.783	Scale	3.875	Form	3.377
	Scale	2.198	Standard dev.	1.193	Lower Threshold	0.096	Scale	4.196
February	Form	7.093	Medium	3.431	Scale	3.518	Form	3.350
	Scale	2.067	Standard dev.	1.127	Lower Threshold	0.098	Scale	3.812
March	Form	6.628	Medium	3.375	Scale	3.485	Form	3.068
	Scale	1.964	Standard dev.	1.184	Lower Threshold	0.098	Scale	3.767
April	Form	5.856	Medium	3.135	Scale	3.250	Form	2.894
	Scale	1.868	Standard dev.	1.147	Lower Threshold	0.094	Scale	3.501
May	Form	4.171	Medium	3.093	Scale	3.318	Form	2.450
	Scale	1.348	Standard dev.	1.323	Lower Threshold	0.051	Scale	3.474
June	Form	3.942	Medium	3.394	Scale	3.659	Form	2.456
	Scale	1.161	Standard dev.	1.444	Lower Threshold	0.032	Scale	3.806
July	Form	5.712	Medium	3.713	Scale	3.863	Form	3.243
	Scale	1.538	Standard dev.	1.240	Lower Threshold	0.054	Scale	4.113
August	Form	5.524	Medium	2.989	Scale	3.103	Form	2.885
	Scale	1.848	Standard dev.	1.108	Lower Threshold	0.090	Scale	3.340
September	Form	3.592	Medium	2.366	Scale	2.589	Form	2.196
	Scale	1.518	Standard dev.	1.128	Lower Threshold	0.036	Scale	2.667
October	Form	3.666	Medium	2.420	Scale	2.640	Form	2.109
	Scale	1.514	Standard dev.	1.195	Lower Threshold	0.067	Scale	2.729
November	Form	5.656	Medium	3.060	Scale	3.185	Form	2.969
	Scale	1.849	Standard dev.	1.095	Lower Threshold	0.069	Scale	3.409
December	Form	6.017	Medium	3.438	Scale	3.591	Form	3.061
	Scale	1.735	Standard dev.	1.227	Lower Threshold	0.093	Scale	3.867

### 3.6 Perspectives of wind and solar technologies in the departments of San Andrés, Providencia, and Santa Catalina

The Colombian department of San Andrés, Providencia, and Santa Catalina presents challenges of energy sustainability due to different factors such as population and commercial growth on the island, high costs of electricity service from fossil fuels, GHG emissions resulting from this generation, increased energy demand, lack of incentives for efficient energy consumption due to existing subsidies [23].

In response to the challenges mentioned above, there is the option of using alternative technologies for energy production, according to the results of this study would be the introduction of distributed energy with photovoltaic solar panels as there are appreciable values in terms of hours of solar brightness and solar radiation, as well as the use of solar thermal technology for the production of domestic hot water (ACS, Agua Caliente Sanitaria). UPME estimates that unconventional energy sources renewable could help reduce thermal generation by 14%. From the perspective of the implementation of wind energy technologies in the archipelago, given the available resource, a wind farm with an installed capacity of up to 7.5 MW can be planned, with approximately five wind turbines that will allow the production of electricity of up to 10 GWh per year [23].

## 4 Conclusions

According to the results obtained in this research, it is noteworthy that the mathematical model used for the study area, the solarimetric information is obtained with a high reliability value. By means of the data provided by the IDEAM, the radiation parameters that allow establishing opportunities for the implementation of solar technologies were calculated by giving the great potential of the study area.

In this research, it was possible to determine by means of the Bird & Hulstrom model and standardizations of the Mächler & Iqbal model, that the Archipelago of San Andrés, Providencia, and Santa Catalina present average total annual radiation values of 6.5 kWh/m^2^ per day, of which 65% represents direct radiation and 35% diffuse radiation. In the department of San Andrés, Providencia and Santa Catalina it is possible to implement solar electric technologies such as photovoltaic and thermal type ACS, since it has great solar potential.

As for the wind resource, the department of San Andrés, Providencia, and Santa Catalina has wind speeds between 0.1 m/s and a maximum value of 15.3 m/s, and in October the average wind speed reaches its maximum value with a data dispersion of 1.19532. With this resource, the installation of wind turbines is projected and therefore, it is possible to generate electrical energy of up to 10 GWh per year**.**

Similarly, the implementation of an ANN allows us to conclude that the use of an autonomous learning method is a great option for the prediction of solar irradiation in areas where there is no measurement instrument for this. The correlation coefficient achieved with this method, will allow the use of data such as the wind speed, the ambient temperature, the relative humidity, the pressure and the solar potential could be obtained for possible generation farms in areas with little access or far from large cities.

## Declarations

### Author contribution statement

Oscar Churio Silvera: Conceived and designed the experiments; Performed the experiments; Analyzed and interpreted the data; Wrote the paper.

Marley Vanegas Chamorro & Guillermo Valencia Ochoa: Conceived and designed the experiments; Performed the experiments; Analyzed and interpreted the data; Contributed reagents, materials, analysis tools or data; Wrote the paper.

### Funding statement

This work was supported by the Universidad del Atlántico with the research grant ING33-CII2009 and ING82-CII2009.

### Data availability statement

The data that has been used is confidential.

### Declaration of interests statement

The authors declare no conflict of interest.

### Additional information

No additional information is available for this paper.

## Appendix A Supplementary data

Appendix A Appendix A

Appendix A. WIND DATA RAW SAN ANDRES AND PROVIDENCIA Appendix A. WIND DATA RAW SAN ANDRES AND PROVIDENCIA

Appendix B. Solar Data Raw San Andres Appendix B. Solar Data Raw San Andres
