# Supplementary material for: Wind and solar resource assessment and prediction using Artificial Neural Network and semi-empirical model: case study of the Colombian Caribbean region
Source: Heliyon. 2021 Sep 8;7(9):e07959. doi: 10.1016/j.heliyon.2021.e07959 (PMC8441173; doi:10.1016/j.heliyon.2021.e07959)
Supplement: Appendix A [file mmc1.docx]

Appendix A

**Table A.1**. Statistical analysis of the data from the weather station Apt. Sesquicentenario

| Month | Counting | Average | Standard Deviation | Coefficient of Variation | Min | Max | Range | Standardized Bias | Standardized Curtosis |
| --- | --- | --- | --- | --- | --- | --- | --- | --- | --- |
| January | 8184 | 3.78264 | 1.1925 | 31.53% | 0.1 | 9.6 | 9.5 | 8.76495 | 18.4853 |
| February | 7464 | 3.43088 | 1.12662 | 32.84% | 0.1 | 7.8 | 7.7 | -4.01116 | 3.86084 |
| March | 8184 | 3.37538 | 1.18377 | 35.07% | 0.1 | 7.2 | 7.1 | 7.14694 | 5.88717 |
| April | 7920 | 3.13511 | 1.14659 | 36.57% | 0.1 | 10.3 | 10.2 | 10.0914 | 30.8864 |
| May | 8184 | 3.09344 | 1.32331 | 42.78% | 0.1 | 9.3 | 9.2 | 13.8834 | 8.38565 |
| June | 8184 | 3.7131 | 1.24025 | 33.40% | 0.1 | 7.8 | 7.7 | -10.6878 | 8.83427 |
| July | 8184 | 3.7131 | 1.24025 | 33.40% | 0.1 | 7.8 | 7.7 | -10.6878 | 8.83427 |
| August | 8184 | 2.98915 | 1.10825 | 37.08% | 0.1 | 8.5 | 8.4 | 5.02723 | 7.56969 |
| September | 7920 | 2.36606 | 1.12773 | 47.66% | 0.1 | 7.2 | 7.1 | 19.3777 | 8.26371 |
| October | 8184 | 2.42046 | 1.19532 | 49.38% | 0.1 | 15.3 | 15.2 | 43.5454 | 120.229 |
| November | 7920 | 3.05954 | 1.09538 | 35.80% | 0.1 | 9.3 | 9.2 | 3.86367 | 14.5409 |
| December | 8184 | 3.46765 | 1.22704 | 35.39% | 0.1 | 7.5 | 7.4 | 0.0863647 | 4.25366 |

**Table A.2.** Multi-yearly adjusted distributions for the weather station Apt. Sesquicentenario

| Month | Gamma | | Standard | | Rayleigh | | Weibull | |
| --- | --- | --- | --- | --- | --- | --- | --- | --- |
| January | Form | 8.31457 | Medium | 3.78264 | Scale | 3.875 | Form | 3.37656 |
|  | Scale | 2.19809 | Standard dev. | 1.1925 | Lower Threshold | 0.0956708 | Scale | 4.19582 |
| February | Form | 7.09293 | Medium | 3.43088 | Scale | 3.51778 | Form | 3.34972 |
|  | Scale | 2.06738 | Standard dev. | 1.12662 | Lower Threshold | 0.098364 | Scale | 3.81200 |
| March | Form | 6.6283 | Medium | 3.37538 | Scale | 3.4849 | Form | 3.06847 |
|  | Scale | 1.96372 | Standard dev. | 1.18377 | Lower Threshold | 0.0976741 | Scale | 3.76716 |
| April | Form | 5.85575 | Medium | 3.13511 | Scale | 3.24999 | Form | 2.89444 |
|  | Scale | 1.8678 | Standard dev. | 1.14659 | Lower Threshold | 0.0940667 | Scale | 3.50072 |
| May | Form | 4.17118 | Medium | 3.09344 | Scale | 3.31795 | Form | 2.44971 |
|  | Scale | 1.34839 | Standard dev. | 1.32331 | Lower Threshold | 0.0507641 | Scale | 3.47395 |
| June | Form | 3.94192 | Medium | 3.39447 | Scale | 3.65941 | Form | 2.45621 |
|  | Scale | 1.16128 | Standard dev. | 1.44358 | Lower Threshold | 0.0317802 | Scale | 3.80552 |
| July | Form | 5.71192 | Medium | 3.7131 | Scale | 3.86341 | Form | 3.24296 |
|  | Scale | 1.53831 | Standard dev. | 1.24025 | Lower Threshold | 0.0541558 | Scale | 4.11333 |
| August | Form | 5.52441 | Medium | 2.98915 | Scale | 3.10327 | Form | 2.88518 |
|  | Scale | 1.84815 | Standard dev. | 1.10825 | Lower Threshold | 0.0904986 | Scale | 3.34038 |
| September | Form | 3.59184 | Medium | 2.36606 | Scale | 2.58872 | Form | 2.19577 |
|  | Scale | 1.51807 | Standard dev. | 1.12773 | Lower Threshold | 0.0358511 | Scale | 2.66677 |
| October | Form | 3.66557 | Medium | 2.42404 | Scale | 2.63957 | Form | 2.10858 |
|  | Scale | 1.51441 | Standard dev. | 1.19532 | Lower Threshold | 0.0670102 | Scale | 2.72919 |
| November | Form | 5.65614 | Medium | 3.05954 | Scale | 3.18488 | Form | 2.96909 |
|  | Scale | 1.84869 | Standard dev. | 1.09358 | Lower Threshold | 0.0689296 | Scale | 3.40911 |
| December | Form | 6.01673 | Medium | 3.46765 | Scale | 3.5908 | Form | 3.06067 |
|  | Scale | 1.7351 | Standard dev. | 1.22704 | Lower Threshold | 0.0929854 | Scale | 3.86682 |


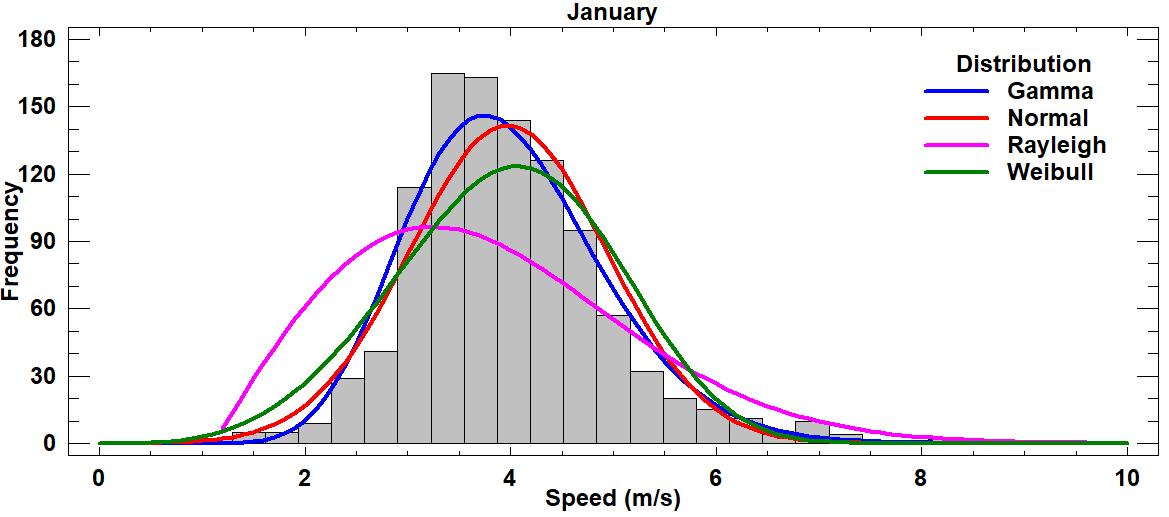


**Figure A**.**1**. Wind speed frequency distributions set for January at the Apt. Sesquicentenario


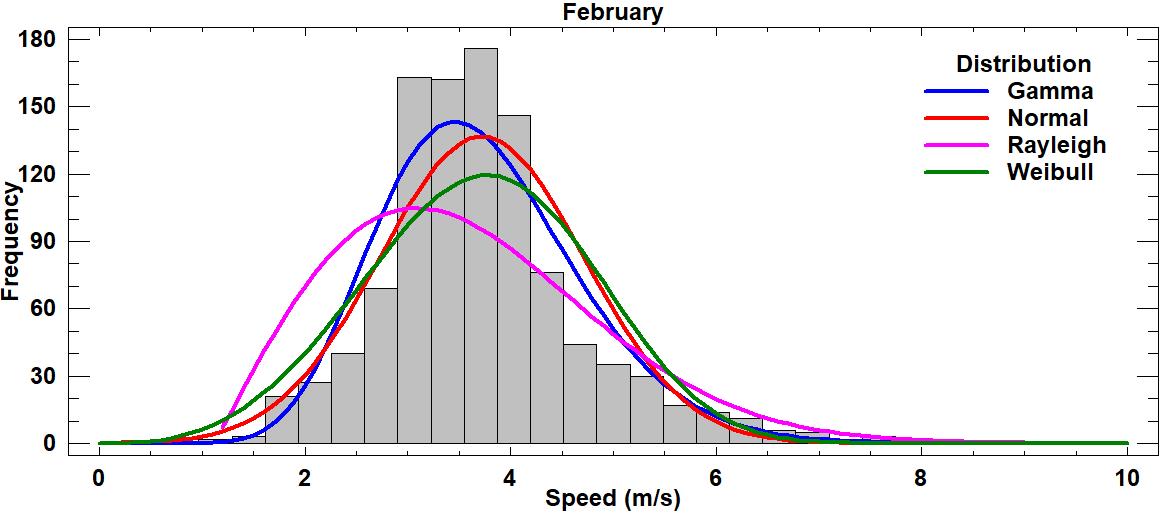


**Figure A**.2. Wind speed frequency distributions set for February at the Apt. Sesquicentenario


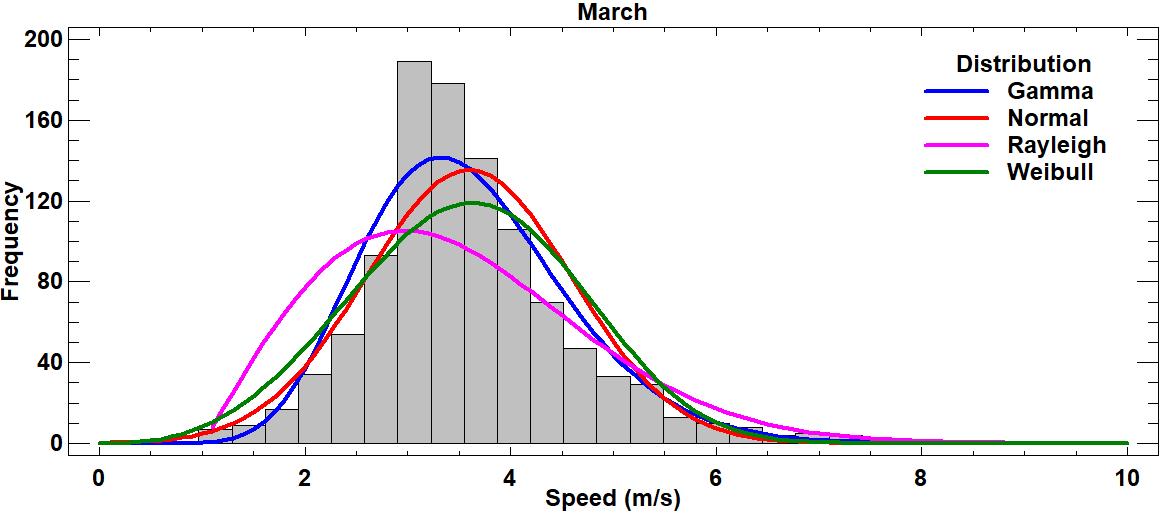


**Figure A**.3. Wind speed frequency distributions set for March at the Apt. Sesquicentenario


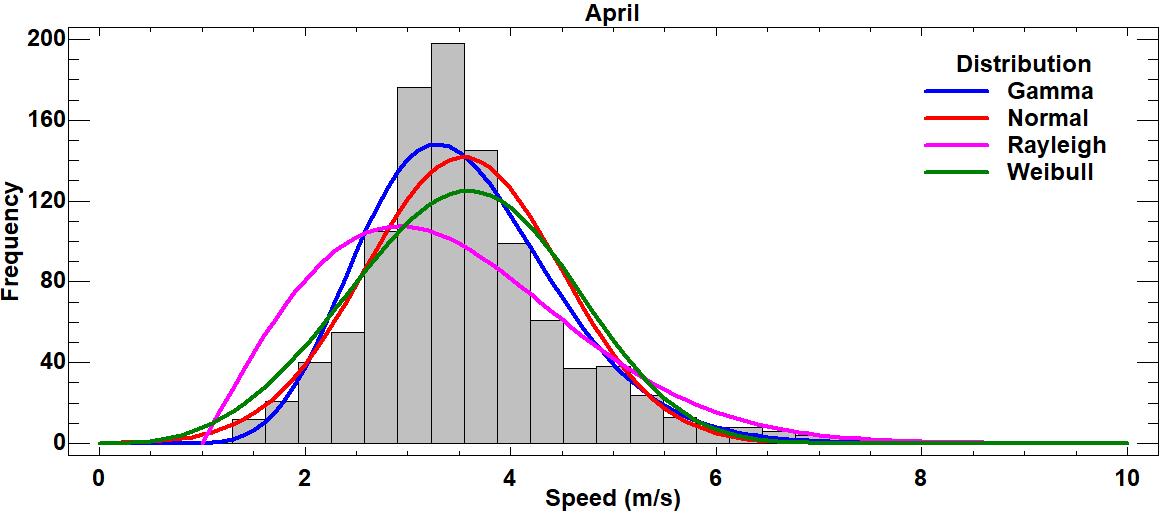


**Figure A.4**. Wind speed frequency distributions set for April at the Apt. Sesquicentenario


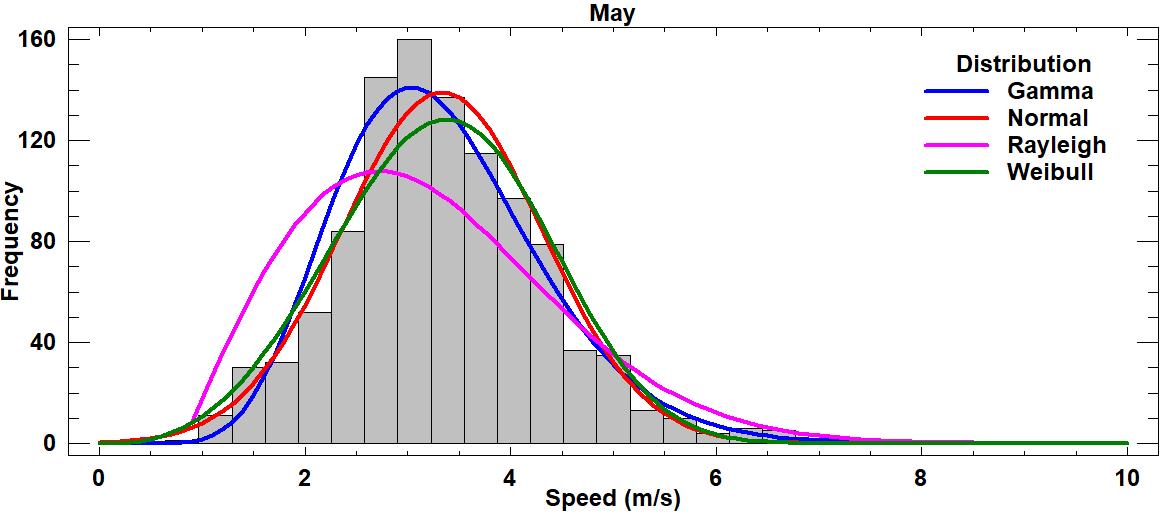


**Figure A.5**. Wind speed frequency distributions set for May at the Apt. Sesquicentenario


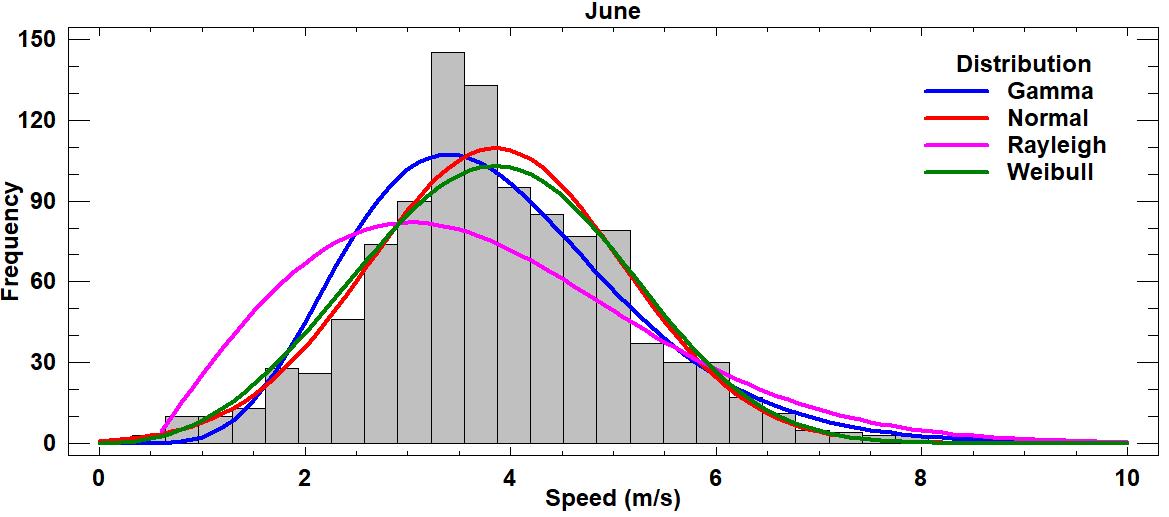


**Figure A.6**. Wind speed frequency distributions set for June at the Apt. Sesquicentenario


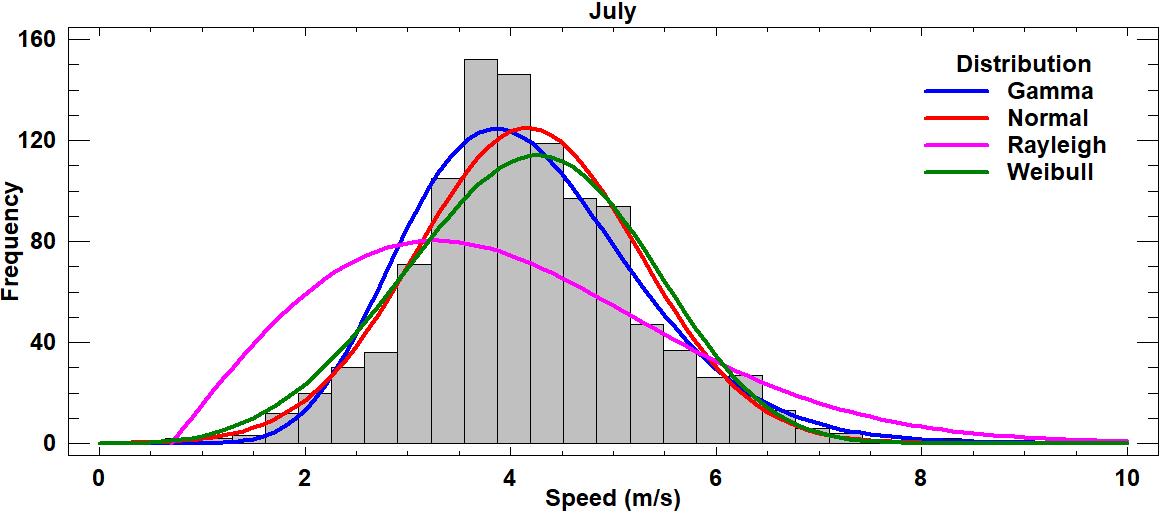


**Figure A.7**. Wind speed frequency distributions set for July at the Apt. Sesquicentenario


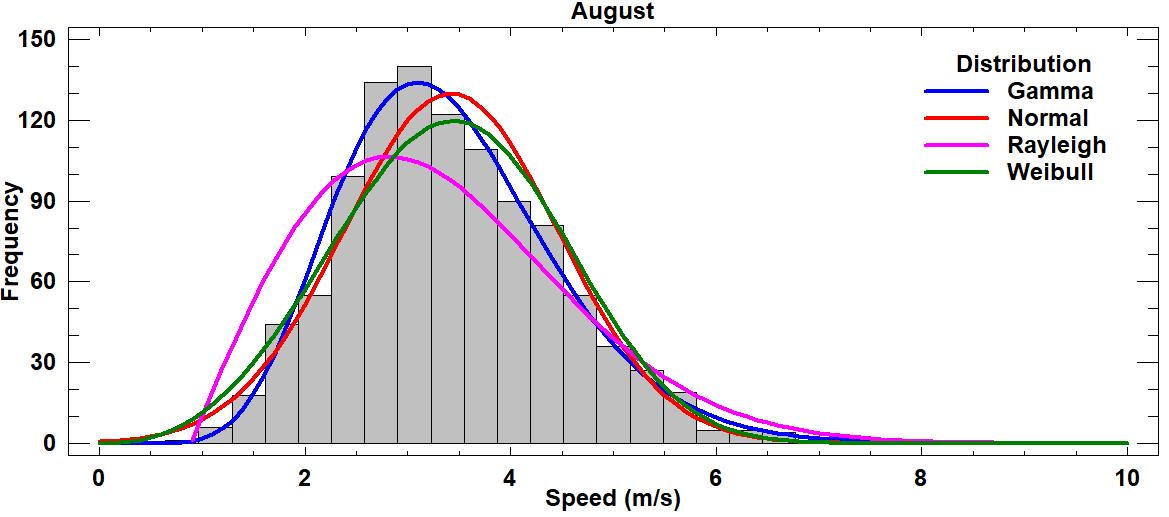


**Figure A.8**. Wind speed frequency distributions set for August at the Apt. Sesquicentenario


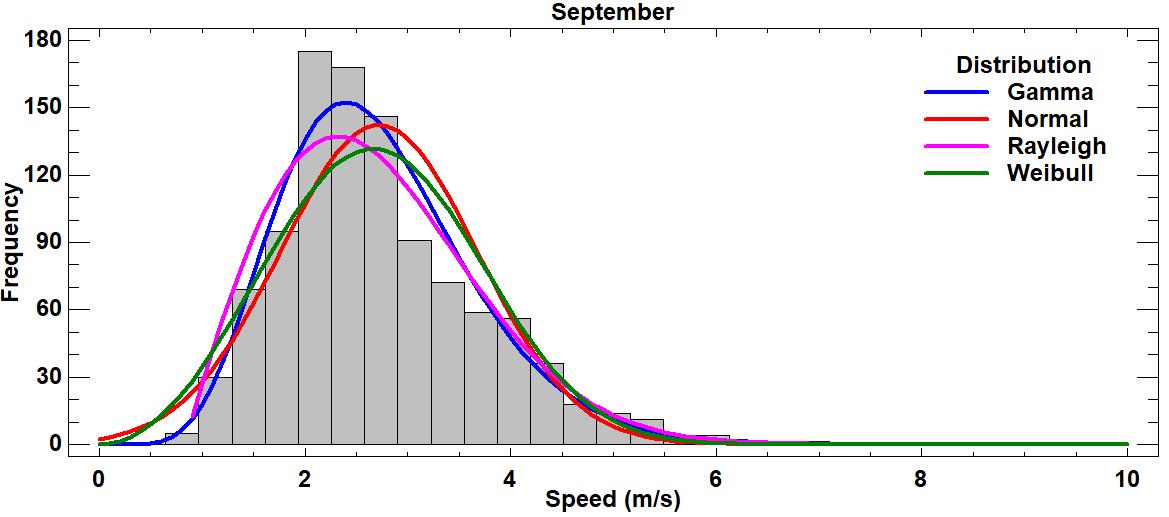


**Figure A.9**. Wind speed frequency distributions set for September at the Apt. Sesquicentenario


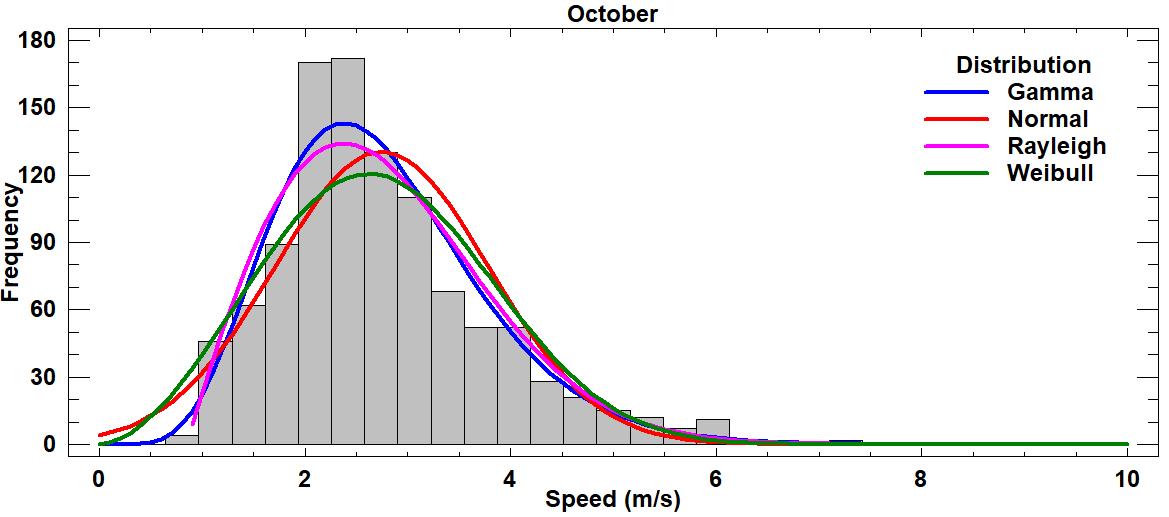


**Figure A.10**. Wind speed frequency distributions set for October at the Apt. Sesquicentenario


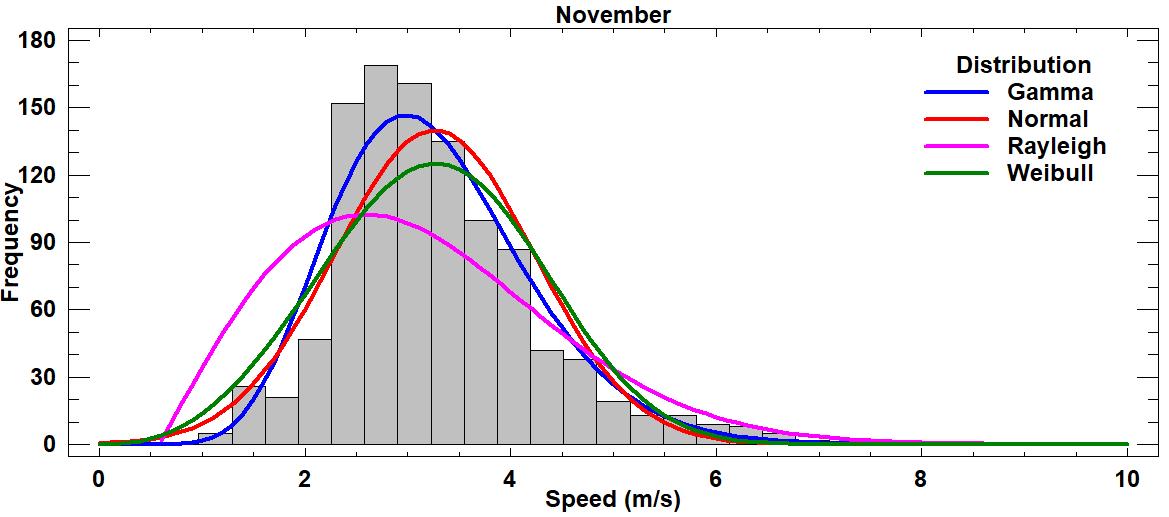


**Figure A.11**. Wind speed frequency distributions set for November at the Apt. Sesquicentenario


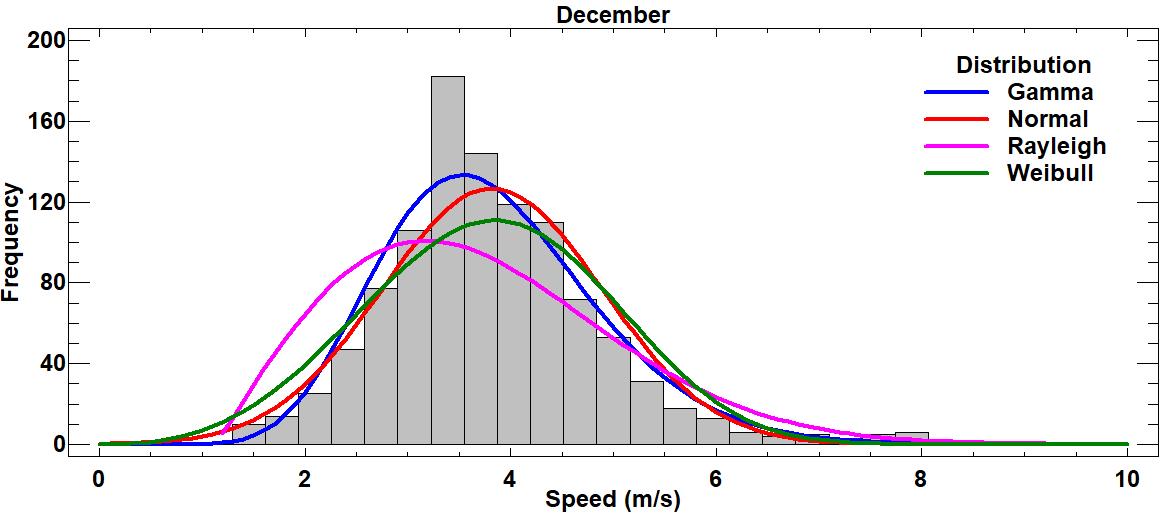


**Figure A.12**. Wind speed frequency distributions set for December at the Apt. Sesquicentenario
